# Nutritional Risk Factors, Microbiota and Parkinson’s Disease: What Is the Current Evidence?

**DOI:** 10.3390/nu11081896

**Published:** 2019-08-14

**Authors:** Christa Boulos, Nathalie Yaghi, Rita El Hayeck, Gessica NHA Heraoui, Nicole Fakhoury-Sayegh

**Affiliations:** 1 Department of Nutrition, Faculty of Pharmacy, Saint-Joseph University of Beirut, Damascus Road, P.O. Box 11-5076, Riad el Solh, Beirut 1107 2180, Lebanon; 2 Department of Geriatrics, Faculty of Medicine, Saint-Joseph University of Beirut, Damascus Road, P.O. Box 11-5076, Riad el Solh, Beirut 1107 2180, Lebanon

**Keywords:** Parkinson’s disease, neurodegenerative diseases, dietary factors

## Abstract

Parkinson’s disease (PD) is a frequent neurodegenerative disease among elderly people. Genetic and underlying environmental factors seem to be involved in the pathogenesis of PD related to degeneration of dopaminergic neurons in the striatum. In previous experimental researches oxidative stress, mitochondrial dysfunction, homocysteine, and neuroinflammation have been reported as potential mechanisms. Among environmental factors, nutrition is one of the most investigated areas as it is a potentially modifiable factor. The purpose of this review is to provide current knowledge regarding the relation between diet and PD risk. We performed a comprehensive review including the most relevant studies from the year 2000 onwards including prospective studies, nested case-control studies, and meta-analysis. Among dietary factors we focused on specific nutrients and food groups, alcoholic beverages, uric acid, and dietary patterns. Furthermore, we included studies on microbiota as recent findings have shown a possible impact on neurodegeneration. As a conclusion, there are still many controversies regarding the relationship between PD and diet which, beside methodological differences among studies, may be due to underlying genetic and gender-specific factors. However, some evidence exists regarding a potential protective effect of uric acid, poly-unsaturated fatty acids, coffee, and tea but mainly in men, whereas dairy products, particularly milk, might increase PD risk through contaminant mediated effect.

## 1. Introduction

**Figure 1:**
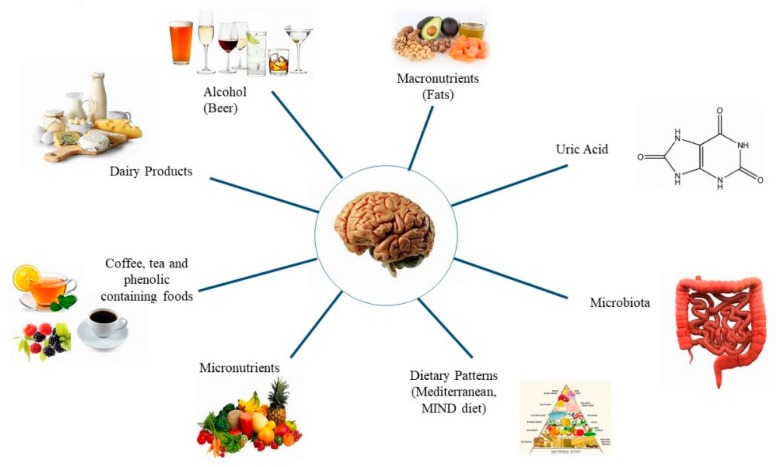
Factors studied in relation to Parkinson’s disease.

Parkinson’s Disease (PD) is a very common neurodegenerative disorder with an incidence of 10 to 18 per 100,000 persons-years [1]. The disease is more prevalent in men than in women [2]. The main signs of PD include bradykinesia, which is the cardinal symptom, muscular rigidity, rest tremor, and gait impairment. The characteristic pathological finding associated with the motor signs of PD is degeneration of the dopaminergic neurons of the pars compacta of the substantia nigra, resulting in loss of dopamine in the striatum [3]. Lewy bodies—eosinophilic inclusion bodies containing alpha-synuclein (αSYN)—are present mainly in the surviving neurons and are considered as the biological marker of neuronal degeneration in PD [3]. The etiology of PD involves both genetic and environmental factors. Among the latter, interest has been growing in the influence of food and nutrients on the development of PD. Furthermore, evidence suggests that possible modification of the gut microbiota may be involved in the pathogenesis of PD, inducing immune cell activation and neuroinflammation of the central nervous system [4]. In this review, we summarize the most important findings based on epidemiologic studies investigating the potential role of dietary factors and microbiota on the development of PD (Figure 1).

## 2. Materials and Methods

Our research was based on the Medline database through its search engine Pubmed. We used the following Medical Subject Headings (MeSH) terms or textwords: “Parkinson’s disease” and “risk factor/s” and “food” or “diet” or “dairy products” or “milk” or “dietary fats” or “fatty acids” or “fatty acids, unsaturated” or “lipids” or “cholesterol” or “vitamins” or “micronutrients” or “antioxidants” or “metals” or “uric acid” or “polyphenols” or “ethanol” or “alcohol drinking” or “caffeine” or “coffee” or “tea” or “Mediterranean diet” or “microbiota”.

Studies included in this review: Human studies, studies published in English language, date of publication from 2000 onwards, prospective studies, meta-analyses, and nested case-control studies. The exception was for microbiota where we included also single case-control studies due to the absence of prospective studies.

Studies excluded: Cross-sectional studies, single case-control studies (except for microbiota), animal and in vitro studies, non-English language articles, studies published before 2000, studies investigating the association between plasma levels of nutrients and PD risk, effect of vitamin and/or mineral supplementation alone on PD risk. Furthermore, relation between PD risk and vitamin D intake was not studied in this review as vitamin D status is mainly determined by sun exposure.

To avoid the possibility of missing articles due to the search strategy, we also performed a manual screening of the reference list of the retrieved articles. For precision purposes, all relevant articles were reviewed independently by two researchers to check for any discrepancies and to ensure rigorous data extraction. For each article, we focused on the key study characteristics, including publication year, country of origin, study design, sample size, and participant characteristics. Studies included in this review are summarized as supplementary material (Supplementary Tables S1–S9).

**Figure 2:**
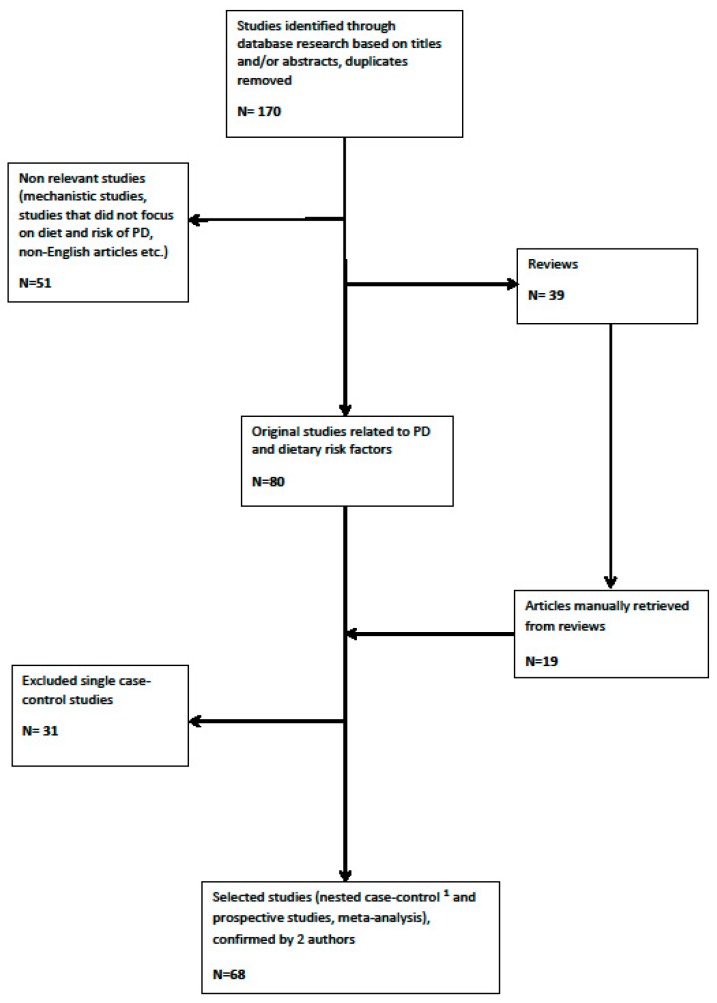
Flowchart of study selection (^1^ except for microbiota).

Details of the study selection process are illustrated in Figure 2.

The review is divided according to the main factors that might be associated with the onset of PD: Nutrients, uric acid, specific food, dietary patterns, and microbiota. In each section, we first discuss results from nested case-control studies (if available), followed by prospective studies, and finally meta-analyses. At the end of each section, findings are discussed whereas recommendations for future research are provided in the conclusion.

## 3. Results

### 3.1. Nutrients

Among the different nutrients, we considered in this review those who were mainly studied in relation to PD risk, such as fats and different kind of fatty acids, vitamins, and minerals.

#### 3.1.1. Dietary Fats

In vitro and animal models showed that cholesterol, oxysterols and other lipids, such as saturated fatty acids (SAFA) have been implicated in PD pathogenesis. Possible mechanisms are: Modulation of α-SYN aggregation, a major constituent of Lewy bodies, destruction of dopamine containing neurons, increase of oxidative stress, and regulation of the production of pro/anti-inflammatory cytokines, all being possible contributors to PD [5,6,7,8,9,10].

A total of ten studies were considered in this review.

In the Farming and Movement Evaluation (FAME) study, a nested US case-control study, an inverse association was found between intake of polyunsaturated fatty acids (PUFA), omega 3 polyunsaturated fatty acids (N-3 PUFA), and α-linolenic acid (aLNA) with PD in dose–response trends [11]. Among western population (Europe, North America), PUFA was found to be inversely associated with PD risk in only two of the four reviewed prospective studies [12,13,14,15]. In the U.S., the two cohorts (2003 and 2014), failed to find an association between total PUFA and PD risk [12,15]. As an individual fatty acid (FA), arachidonic acid (AA) was the only one that tended to be inversely associated with the risk of PD in the first cohort, whereas in the second cohort, only omega 6 polyunsaturated fatty acids (N-6 PUFA), and linoleic acid (LA) showed a positive association with PD risk in both genders [12,15]. Furthermore, in a Greek cohort of the European Prospective Investigation into Cancer and Nutrition (EPIC) study, the authors reported an inverse association between total PUFA intake and incidence of PD (*p* = 0.032). After additional adjustments for smoking and vitamin E intake, a significantly decreased risk of PD was also observed for the intake of N-6 PUFA, (HR: 0.69; 95% CI: 0.47–1.00). Among the PUFAs, only aLNA and LA were significantly associated with a lower risk of PD [14].

In the U.S., in the first cohort, replacing PUFA with SAFA in 5% of the energy intake significantly increased the risk of PD in men only [12].

Regarding intake of both total fat and monounsaturated fatty acids (MUFA), only de Lau et al., in the Rotterdam study, found a significant association with a lower PD risk [13].

In the Honolulu-Asia Aging Cohort Study (HAACS), intake of PUFA appeared to be protective particularly in men who never smoked cigarettes (*p* = 0.042). No association was found with dietary cholesterol [16]. Inversely, the Singapore Chinese Health Study (SCHS) showed that higher intakes of cholesterol and MUFA may reduce risk of PD in men and women. However, there was no statistically significant association between dietary intake of SAFA, N-3 PUFA, and N-6 PUFA, and PD risk [17].

We reviewed three meta-analyses; the first was performed by Kamel et al. in 2014 including nine studies (three case-control studies and six prospective studies). Total fat intake was inversely associated with PD, as well as MUFA, SAFA, PUFA, aLNA, and LA. The inverse association was strongest for aLNA and weakest for SAFA. Interestingly, a greater increase in PD risk was associated with pesticide use in individuals with lower intake of PUFAs or higher intake of saturated fat [11].

The second meta-analysis, including data from six prospective and seven case-control studies, did not find any association between total fat intake and PD [18]. Regarding specific FA, a lower risk of PD was reported with increased intake of total PUFA, N-3 PUFA, N-6 PUFA, and LA in prospective studies only, whereas no association was found between cholesterol intake and PD [18].

Similarly, an inverse association was found between overall PUFA and PD, in a meta-analysis performed by Zhang et al. An 8g/day increase in PUFA was found to be associated with a decrease in PD risk, but this association was not found for 0.1 g/day increment of docosahexaenoic acid (DHA) nor with 0.05 g/day increment of eicosapentaenoic acid (EPA) and aLNA [19].

In conclusion, results from cohort studies and meta-analyses tend to pinpoint a protective effect of PUFA, but dietary factors might have a lesser impact after adjusting for confounding factors, such as smoking and caffeine intake [14]. A possible negative effect of N-6 PUFA and protective effect of N-3 PUFA could be linked to their role in the inflammatory pathway [20]. Another explanation of the positive effect of PUFA on PD risk might be related to the presence of fat soluble vitamins such as vitamin E, along with vegetable sources of fat, which has been found to be protective against PD [21,22]. Furthermore, the quality of fat usually consumed across countries is variable, potentially contributing to such contradictory results. 

Further studies would help understand better the impact of dietary fat on PD risk compared to other risk factors.

#### 3.1.2. Vitamins

The vitamins studied in relation to PD have mainly included B-vitamins and antioxidant vitamins such as vitamins C, E, and beta-carotene. We did not consider vitamin D as it is mainly determined by sun exposure and less by dietary intake.

##### B-vitamins

Vitamins B6, B9, and B12 are important cofactors in homocysteine (HC) metabolism. Reduced intake of one or several of these vitamins results in an increased HC level, which is known to have neurotoxic effects [23]. High HC concentration has been described in patients with PD as well as in other neurodegenerative disorders [24]. However, it remains unclear whether these finding are a consequence of changes in dietary habits or medication, especially L-Dopa [25], or if higher HC levels are involved in the pathogenesis of PD [26]. In animal studies, administration of HC into the brains of rats significantly reduced the dopamine level and locomotor function [27]. In addition, subjects with the genotype 677 TT of Methylenetetrahydrofolate reductase (MTHFR), resulting in an impaired folate metabolism and consecutive hyperhomocysteinemia, showed increased risk of PD [28]. Regarding vitamin B6, it converts homocysteine into cysteine, which is a precursor of glutathione (GSH), a major antioxidant in the human body [29]. The presence of GSH in neurons prevents oxidative stress, and low levels of intracellular GSH will lead to apoptosis [30]. Furthermore, vitamin B6 is also a cofactor necessary for the synthesis of dopamine [31]. 

We reviewed two prospective studies carried out among western population. 

De Lau et al. [2] studied the intake of vitamins B6, B9, and B12 in a 10-year prospective study (the Rotterdam cohort). No association was found between both vitamin B9 and B12 intake and PD, whereas increased vitamin B6 intake was related to reduced risk of developing PD (highest vs. lowest tertiles, RR: 0.46; 95% CI: 0.22–0.96). Stratified analyses showed that this association was only present among smokers. In fact, smoking has been found to be related to decreased risk of PD in some studies; however, many controversies related to this topic remain [32]. 

Results from the Nurses’ Health Study (NHS) and the Health Professionals Follow-up Study (HPFS) published by Chen et al. [26] failed to show any association between vitamins B6, B9, and B12 intake and PD incidence after 12.7 years of follow up in men and 17.3 years of follow up in women. The authors explained these findings by the fact that health professionals may have more adequate dietary intakes, which could explain the lack of association. Furthermore, food fortification in North American countries and genetically determined variations in vitamin B metabolism, especially folate, could be additional explications [26].

##### Vitamin C, E, A, and Beta-Carotene 

Vitamins with antioxidant properties have been extensively studied given their ability to reduce cell damage caused by free radicals as a result of aging and other environmental influences. Oxidative stress has been reported to be one of the potential mechanisms participating in death of dopaminergic neurons, which has been supported by several rodent models and postmortem brain analysis [33]. In animal and in vitro-studies, vitamin C has shown its beneficial effects on the differentiation of undifferentiated brain cells into dopaminergic cells and the synthesis of L-Dopa [34]. As for vitamin A, the dopaminergic system is one of the best-established targets of retinoic acid in the brain [35]. 

A total of four studies, one nested case-control study and three prospective studies were included in this review. 

A case-control study nested within the Leisure World cohort study, including 13,979 residents and conducted in Southern California, failed to show an association between intake of vitamin A and C and PD after adjusting for the main covariates [36]. Similarly, no association between antioxidant intake (vitamins C, E, carotenoids) and incidence of PD was reported by Hughes et al. in 2016 in both the HPFS and the NHS [37]. These findings are in contrast with those published in 2002 by Zhang et al. within the same cohort, where a reduced risk of PD was observed when comparing the highest versus the lowest quintile of dietary vitamin E intake (RR: 0.68; 95% CI: 0.49–0.93). In contrast, no association was found for vitamin E from supplements, vitamin C, and carotenoid intake [21]. 

Yang et al. studied the association between dietary intake of vitamins E, C, and beta-carotene in two Swedish population-based cohorts including more than 80,000 men and women. Beta-carotene intake was associated with a reduced incidence of PD in both genders. Vitamin E intake was associated with a 30% reduced risk of PD in women whereas in men, vitamin E was only associated when analyzed as a continuous variable. Vitamin C intake showed a borderline association with PD risk in women only (*p* = 0.04). Following the authors’ explanation, the observed gender differences could be related to hormonal influences, as estrogens may have a preventive action against PD [22].

We identified two meta-analysis, the first was published by Etminan et al. in 2005 and based on six case-control and one cohort study [38]. The authors reported a relative risk reduction for PD of 19% for moderate vitamin E intake (RR: 0.81; 95% CI: 0.67–0.98). However, no association was observed between PD risk and vitamin C and/or beta-carotene intake. The second meta-analysis investigating the relationship between dietary intake of the different types of carotenoids and PD failed to show any significant association [39].

Based on the above findings, evidence is still lacking regarding the potential impact of vitamin intake and PD risk. Among antioxidant vitamins, only vitamin E intake was associated with a reduced risk of PD in three of the four studies. As for B-vitamins, genetic variations in the activities of key enzymes involved in homocysteine metabolism, such as MTHFR, may be one of the main explanations of these variable outcomes. Moreover, fortification of staple food with B-vitamins is currently practiced in the U.S. and serum levels of these vitamins are in general higher in American compared to European population [2]. 

Finally, conducting future prospective studies that investigate the association of PD risk with plasma concentrations of homocysteine, folate, vitamin B6, and/or vitamin B12, and related dietary intakes may be of value. In addition, vitamin B6 may be a good candidate for additional studies due to its multiple roles in homocysteine metabolism, glutathione and dopamine synthesis. 

#### 3.1.3. Minerals 

Limited data are available regarding the relationship between mineral intake and PD risk. We only identified two cohort studies investigating the association between calcium intake and PD and one cohort study and one meta-analysis studying the intake of iron and/or other trace elements and PD risk.

##### Calcium

Calcium has multiple functions in the human body. Beside the structural role in bone tissue, calcium is necessary for many cell processes including ion channel activation and second messenger activity [40]. Based on mechanistic studies, the elevation in cytosolic Ca^2+^ following abnormal calcium channel activity could be one reason for the death of dopaminergic neurons. High concentration of intracellular calcium promotes aggregation of αSYN, a major component of Lewy bodies [41]. 

Results from both U.S. cohorts (HPFS and NHS), including 47,331 men and 88,563 women, showed an 50% increased risk of PD when comparing the highest with the lowest quintile of dairy calcium intake in men (*p* = 0.02) after adjusting for potential confounders. However, no association was found for non-dairy calcium intake and risk of PD [42]. 

Another study was conducted in Hawaii within a population of Japanese origin, aiming to examine the relationship between dairy products, calcium intake and PD risk [43]. The study was part of the Honolulu Heart Program and included 7,504 men with 30 years of follow-up. Quartiles of calcium intake was studied from dairy and non-dairy sources. Similar to the finding from the U.S. cohorts, incidence of PD increased with higher intake of calcium from dairy sources (*p* = 0.046) but not from non-dairy sources (*p* = 0.704). After adjustment for milk, calcium intake had no influence on PD incidence. Hence, as previously, the observed association between dietary calcium and PD is explained by concomitant milk intake and “may be confounded by other unmeasured constituents or contaminants” of dairy products [42].

##### Iron and other Trace Elements

Iron is an essential trace element involved in various physiological functions. However, free iron can catalyze the production of reactive oxygens species (ROS) and therefore increase oxidative stress that may damage membranes and other cell structures. In addition, there is some evidence for other mechanisms unrelated to oxidative damage, such as apoptosis and interaction with abnormal protein aggregates [44]. Several studies have reported increased iron deposits in the area of the substantia nigra in PD patients [45]. Long-term occupational exposure to specific metals, including iron, was found to be related to increased risk of PD; however, whether iron intake from other sources, such as from food and supplements, may have the same effects needs to be clarified [46]. Furthermore, other essential trace elements, such as zinc and copper, may be involved in the pathogenesis of neurodegenerative diseases through their antioxidant properties.

Logroscino et al. reported results from the U.S. health professionals’ cohorts (HPFS and NHS). After a mean follow up of 15 years, no association was found between total iron intake (food and supplements) and PD incidence when comparing extreme quintiles and after adjusting for other covariates [47]. However, a nearly 30% increased risk was found for dietary non-heme iron (*p* = 0.02), but not for heme iron. This association was stronger among subjects with low vitamin C intake. The increased risk for PD was observed in both cohorts and was mainly due to consumption of a high amount of iron-fortified grains and cereals. The authors hypothesized that there might be a dysregulation of absorption with a less efficient control of intestinal absorption and entry of iron into the central nervous system. Notably, the average intake of iron in both cohorts was more than twice the recommended daily intake [47]. Other essential trace elements, such as zinc and copper, may be involved in the pathogenesis of neurodegenerative diseases through their antioxidant properties as supported by several studies [48,49]. 

Cheng et al. investigated the relationship between dietary intake of iron, zinc, and copper and PD risk in a recent meta-analysis [50]. The authors found that the risk of PD was increased in western population (OR: 1.47; 95% CI: 1.17–1.85) and in men (OR: 1.43; 95% CI: 1.01–2.0) through high Fe intake, whereas no association was reported within the total population. In addition, no association was found for copper and zinc intake and PD risk. Following the authors comments, a possible explanation may be ethnicity and gender specificities as, first, PD is more prevalent in men than in women, and second, women have lower iron stores due to menstrual blood loss [50].

In conclusion, there is some evidence linking high non-heme iron intake to increased PD risk. However these findings should be considered with caution. Further investigations studying biomarkers of oxidative stress, iron status, and homeostasis in combination with iron intake from different sources (food and supplements, heme and non-heme iron) would be of interest to confirm these results and clarify the underlying mechanism.

### 3.2. Uric Acid

Uric acid (UA) is a physiological end product of purine synthesis that has strong antioxidant properties [51]. UA has also metal-binding properties, preventing toxic effects of iron and copper on neurons [52]. The plasma levels of urate are determined by dietary intake, renal excretion, and genetic factors that influence urate metabolism [53]. Studies analyzing postmortem tissue showed low urate levels in the substantia nigra of Parkinson’s patients [54]. In animal models, UA showed neuroprotective effects on dopaminergic neurons through modulation of neuroinflammation and oxidative stress [55].

We reviewed six cohort studies investigating the relationship between urate concentration and incidence of PD. In most studies, plasma urate level was analyzed by quartiles.

De Lau et al. reported findings from the Rotterdam study, a population-based cohort including nearly 5000 participants aged 55 years and above. After a mean follow up of 9.4 years, a higher UA concentration was independently related to a significantly lower risk of PD (OR: 0.42; 95% CI: 0.18–0.96) in both gender, with evidence for a dose–effect relationship [56]. In addition, within a U.S. cohort based on the Atherosclerosis Risk in Communities (ARIC) study, after a mean follow up of 20 years, serum UA level was inversely associated with PD incidence within the total population and in men after adjusting for numerous potential confounders [57].

Similar results emerged from the U.S. HPFS cohort, where a significant reduction in PD incidence was observed with increasing urate quartiles (RR = 0.43; *p* = 0.017) [58]. However, these studies did not provide a proof that UA is a protective factor against PD. That’s why based on the same cohort, Gao et al. [59] designed a study investigating whether a diet that increases plasma urate level is also related to a decreased PD incidence. When comparing the extreme quintiles of the dietary urate index, the relative risk of PD was significantly reduced. These important findings, based on diet induced changes in serum urate level, add additional evidence to the fact that UA or its metabolites may have a protective effect on PD risk [59].

In addition two recent meta-analyses, the first published in 2013 [60] and the second in 2017 [61] showed that, in both Asian and non-Asian populations, plasma urate levels were significantly lower in PD patients compared to age-matched healthy control subjects. When comparing the urate levels between early stage and middle-late stage disease, the authors found a decrease with the progression of the disease, suggesting that UA might act as potential biomarker indicating the risk and the progression of the illness rather than as a risk factor by itself [61].

Many studies failed to show an association between urate levels and PD in women. For example, findings from a nested case-control study conducted in England reported a 30% reduction in PD in men with a previous history of gout but not in women [62]. Similarly, a case-control study nested within the female U.S. NHS cohort reported no difference in urate levels between cases and controls regardless of potential confounders [63]. These findings were confirmed by Jain et al. [64] among a U.S. population who, after a follow up of 14 years, reported a significant association between the low urate level compared to the mid-range urate level category with a nearly 1.7-fold increased risk of PD (*p* = 0.04) in men, after adjustment for confounders. Gao et al., within three ongoing U.S. cohorts, found that a higher urate concentration was significantly associated with a lower risk of PD in men only [65]. Similar results were obtained through a meta-analysis performed by the same authors reporting a relative risk of 0.63 (95% CI: 0.42–0.95) with increased urate levels in men but no association in women [65]. 

In conclusion, studies have consistently reported an association between higher urate levels and lower PD risk. However, whether this association is causal remains still unclear and needs further investigations in addition to the interesting study done by Gao et al. highlighting the protective effect of UA on PD risk through dietary intervention [59]. 

Furthermore, associations were mainly present in men compared to women. Whether these findings reflect a real difference in the underlying mechanisms or if they are due to lack of statistical power related to the lower urate concentration and PD incidence in women needs still to be clarified [65]. In fact, beside the positive effect on urate excretion, estrogens have also a neuroprotective effect [60].

Finally UA seems to have a real potential to reduce the progression of the disease as shown by Ascherio et al. who reported a significant association between serum and brain urate level and rate of clinical decline in PD [66]. The authors “established urate as the first molecular predictor of clinical progression in PD and provided a rationale for investigating the possibility that a therapeutic increase in urate in patients with PD might act favorably to slow down the disease course” [66].

### 3.3. Specific Foods 

Few studies reported data related to intake of specific foods and risk of PD. Those are mainly dairy products, alcoholic beverages, tea, coffee and other polyphenol-rich foods.

#### 3.3.1. Dairy Products

Several in vitro and animal model studies have noted the potential neurotoxic effect of milk and dairy products, either through increasing oxidative stress via intracellular calcium effect or through contaminants like rotenone and paraquat that act as toxic substance in nigrastriatal neurons [67,68].

In this review, seven cohorts were found that investigated the link between dairy products and PD risk.

In 2002 in the U.S., the first results of two large prospective cohorts, the NHS and the HPFS, showed a strong association between PD risk and consumption of dairy products in men, but not in women. Compared with those who consumed less than one serving per day, men who consumed 2.9 or more servings per day had an 80% increase in risk of PD [42]. Calcium and vitamin D intake were associated with PD only when the source was dairy, suggesting that other confounding factors or contaminants might be involved [42,69,70]. Similar results were found by Chen et al. in the American Cancer Prevention Study II Nutrition Cohort (CPSII) after a 9 years follow up [70].

In 2017, Hughes et al. presented updated results from the NHS and HPFS cohorts, with 26 and 24 years of follow up, respectively. Results showed a significant association between low fat dairy intake and PD risk but not with total dairy intake. In both cohorts, fat from dairy sources was not associated with PD risk [71], meaning that fat would not be responsible for the observed effect. 

In the Honolulu Heart Program (HHP), data showed a 2.3-fold increased risk of PD in the highest milk intake group versus those who consumed no milk independently of the intake of calcium. Whether the observed effects were mediated through nutrients other than calcium or through neurotoxic contaminants has been questioned [43].

Abbott et al. investigated the relationship between midlife milk intake and PD incidence through associations with substantia nigra neuron density and organochlorine pesticide exposure in post-mortem brain tissues. Detection of heptachlor epoxide was associated with milk intake only in nonsmokers. Milk consumption increases the risk of PD only among non-cigarette smokers suggesting a protective effect of smoking [67].

In Greece within the cohort of the EPIC study, dairy product intake, especially milk consumption, showed a positive association with PD. Conversely, the other dairy products common in the Greek diet, namely, yogurt and cheese, did not exhibit significant associations [14].

In the Finnish Mobile Clinic Health Examination Survey (FMC) conducted over a 41-year follow-up, milk consumed by women showed positive associations with PD risk. Furthermore, intake of reduced-fat dairy products were associated with increased PD risk [72]. 

A recent meta-analysis published by Jiang et al. including five studies performed in Greece, Finland, and the U.S. asserted previously documented links between dairy food intake and increased risk of PD in men. The risk of PD increased by 17% for every 200 g/day increase in milk intake [42,43,70,72,73]. This meta-analysis was considered by Bellou et al. in 2016 as suggestive evidence (class III association), in the umbrella of meta-analysis on that same topic [74].

In summary, epidemiological findings suggest that the frequently reported association between dairy products and PD is unlikely due to calcium, vitamin D, or fat but possibly to other factors such as contamination with neurotoxic pesticides and/or the urate lowering effect of dairy products [43,72,75]. Nevertheless, limiting milk consumption with such level of evidence should not be recommended to prevent PD [76]. Given the more significant association in men than women, further investigations need to be conducted.

#### 3.3.2. Alcoholic Beverages

Several epidemiological studies have investigated the association between alcohol consumption and PD risk. Results from different studies on alcohol intake and PD risk are not consistent; both positive and negative associations as well as no association have been reported.

No associations between alcohol and PD were found in reviewing three case-control studies nested within a cohort—the Leisure World cohort study [36] and the Agricultural Health Study [77] both done in the U.S, the General Practice Research Database done in the U.K. [78]—as well as in reviewing 2 prospective studies—the U.S. Cancer Prevention Study II Nutrition Cohort [79] and the Singapore Chinese Health study conducted between 1993 and 2005 [80]. 

When considering the type of beverage, two prospective studies, the NHS and the HPFS [81] and the NIH-AARP Diet & Health Study [82], found that beer drinkers compared to non-beer drinkers had a significant lower risk of developing PD. According to the NHS and the HPFS, consumption of wine or liquor was not associated with the incidence of PD. However, Liu et al. (NIH-AARP Diet & Health Study) found that liquor consumption increases PD risk (*p* < 0.03), whereas increased wine consumption showed a borderline lower PD risk.

In the Swedish national cohort, Eriksson et al. [83] found that heavy alcohol consumption conferred a 38% increased risk of PD in both sexes after a follow-up of 36 years. The highest risk was observed in the lowest age group (≤44 years). Similarly, in the Finnish Mobile Clinic Health Examination Survey, a prospective study, Sääksjârvi et al., found that individuals who consumed <5 g of alcohol daily compared to non-drinkers had a higher risk of PD [84].

We reviewed two meta-analyses. In the first meta-analysis published in 2012, including 22 case-control and two cohort studies, significant negative associations with PD were found for alcohol consumption. The RR/OR (95% CI) in case-controls studies were 0.92 (0.85–0.99), 0.79 in cohort studies (0.65–0.95), and 0.9 in all studies (0.84–0.96) [85]. In the second meta-analysis published in 2014 including 8 prospective, 17 matched case-control, and 7 unmatched case-control studies, the risk of PD linearly decreased by 5% for every additional alcoholic drink per day. The association was significant in all types of studies with a pooled RR of 0.78 (95% CI: 0.67–0.92). A significant negative association was found with beer but not with wine or liquor and for men but not for women [86]. 

Regarding the underlying physio-pathological mechanisms explaining these findings, experimental studies have shown that moderate alcohol consumption has neuroprotective properties. This appears to be linked to the activation of signal transduction processes, which may involve ROS, different key protein kinases, and heat shock proteins [87].

The different results regarding specific types of alcoholic beverages may be explained by other mechanisms involving additional factors other than ethanol itself. Beer is known to increase UA levels because of its high content of purine, which may, in addition to ethanol, have a synergistic effect on urate level [88,89]. An increase in urate level was observed after consumption of whole or non-alcoholic beer [90,91,92]. As mentioned previously, urate has strong antioxidant properties [93,94,95] and has been associated with lower PD [58,59]. The polyphenolic components of red wine, such as resveratrol, have been shown to reduce neurotoxin 6-hydroxydopamine (6-OHDA)-induced toxicity in animal studies [96,97] through its antioxidant and anti-inflammatory properties. Additionally, resveratrol exerts anti-apoptotic actions in brain cells and attenuates mitochondrial impairment induced by certain stressors [98]. It also triggers mitochondrial biogenesis, improving the mitochondria-related bioenergetics status in mammalian cells [98]. Moreover, several experimental cell-based and in vivo animal studies have shown that resveratrol confer neuroprotection by fostering degradation of alpha-synuclein species through activation of autophagy [98]. However, to date, epidemiologic studies have largely failed to report a neuroprotective effect of wine consumption on risk of PD.

#### 3.3.3. Coffee, Tea, and Other Polyphenols Containing Food Items 

Coffee and tea are common sources of caffeine which have been extensively studied in neurodegenerative diseases [99]. Caffeine’s neuroprotective function is attributed to its antagonistic action on adenosine 2A (A2A) receptors in the brain, which are being increasingly targeted as an antiparkinsonian therapy in clinical trials [100]. Another neuroprotective effect of caffeine is that it activates specific neuroprotection signaling pathways and prevents apoptotic cell death in a PD model using human dopaminergic neuroblastoma cells [100]. 

Five large prospective cohort studies were reviewed. A cohort study published by Ross et al. in 2000 examined the association between coffee intake and PD risk with a long follow up. They observed a significant inverse association between coffee intake and PD risk after adjustment for alcohol and smoking [101]. Another 13 years cohort study investigated the relationship between PD and high coffee intake among 29,335 healthy Finnish subjects [102]. The study reported a 60% decreased risk of the disease for coffee intake of ≥5 cups/day. Ascherio et al., in two older prospective cohort studies, reported the same inverse association between coffee consumption and the risk of PD among men only [103,104]. This difference in the role of caffeine as a preventive factor in men only was related to the intake of postmenopausal estrogens among women [103,104], suggesting a possible role of estrogen in hiding the beneficial effect of caffeine. These results were contradicted by another recent study published by Liu et al. who prospectively examined whether caffeine intake was associated with lower risk of PD among 304,980 participants in the National Institutes of Health-AARP Diet and Health Study [82]. The results showed a lower PD risk in both men and women. Adjustment for smoking and its duration did not materially change the results. Subgroup analysis by hormone use among postmenopausal women suggested that caffeine intake was associated with lower PD risk among hormone users, not among never users. However, the statistical test for interaction was not statistically significant [100]. 

A meta-analysis by Costa et al. in 2010 confirmed an inverse association between caffeine intake and the risk of PD, which can hardly be explained by bias or uncontrolled confounding [105]. The results of the cumulative meta-analysis showed that since 2001, the number of studies on this topic nearly doubled with a pooled RR of 0.72 at the end of 2001 and a pooled RR of 0.75 at the end of 2008 (five cohort/nested case-control and nine case-control/cross sectional studies) [105]. This revealed a 25% reduction in the risk of PD among caffeine consumers with an inverse dose–response relationship between the onset of PD and caffeine intake. There was no meaningful heterogeneity between studies.

A negative association with the onset of PD was also reported for other non-coffee sources of caffeine, such as tea. Tea consumption has major interests: Besides containing caffeine, it contains specific polyphenols that can play an important role in delaying the disease. Polyphenols have been reported to inhibit the formation of α-synuclein misfolded aggregates and to reduce mitochondrial dysfunction-induced oxidative stress and inflammatory responses [106]. Green and black teas are rich sources of polyphenols, commonly known as flavonoids or catechins (flavan-3-ols). There are five major catechins in tea leaves, the most abundant of which is epigallocatechin-3-gallate (EGCG), making up as much as 25%–40% of the total catechin content [107]. Green tea is not fermented and the catechins account for 30%–40% of the solid brewed tea. Conversely, black tea is extensively fermented, which contributes to 3%–10% of catechins in the remaining solids [108]. Black tea is estimated to contribute to 60%–84% of dietary flavonoids intake in western populations [109]. 

We found two prospective cohort studies and one meta-analysis that reported an inverse association between black tea and PD. A study by Tan et al., on a cohort of 63,257 Chinese men and women, reported an inverse relationship between black tea consumption and PD risk with an adjusted RR of 0.29 (*p* = 0.0006) between the highest vs. lowest tertile of tea intake, without being confounded by total caffeine intake or tobacco smoking [80]. Ingredients of black tea other than caffeine appear to be responsible for the inverse association with PD [80]. These results are in line with a previous cohort study, published in 2001, that demonstrated an inverse association between tea consumption (RR: 0.6; 95% CI: 0.3–1.2) and PD risk in a large population of men, after excluding coffee drinkers [104]. Data from Chow et al. also confirmed that a daily dose of 800 mg caffeine-free EGCG for four weeks is safe and well-tolerated in healthy human subjects [110].

These results were further confirmed by a meta-analysis published by Li et al., which included 1418 cases and 4250 controls (seven case-control studies and one cohort study). They reported that tea drinking can lower the risk of PD by 15%, which suggests the protective effect of tea consumption on PD risk with no apparent dose–response relationship [111].

The antioxidant activity of polyphenols is reflected in the activation of nuclear factor erythroid-related factor 2 (Nrf2) pathway (role of Nfr2, ma Q) which regulates neuronal cells antioxidant defense and the consequent upregulation of detoxification enzymes such as heme oxygenase 1 known to regulate inflammatory processes [112]. Many studies showed that phenolics from *Olea europaea* found also in extra virgin olive oil exert strong antioxidant properties and can counteract oxidative stress in brain tissue [113]. The main phenolic subclasses present are phenolic alcohols (tyrosol, hydroxytyrosol), phenolic acids (ferulic acid, vanillic acid, caffeic acid), flavonoids, lignans, and secoiridoids (oleocanthal, oleuropein, ligstroside) [114]. Oleuropein and hydroxytyrosol act as direct free radical scavengers, hydroxytyrosol and oleocanthal are strong cyclooxygenases (COX) inhibitors, and oleuropein counteracts low density lipoprotein (LDL) oxidations [115]. However, studies showing a direct action of phenolic compounds within the brain are rare. Most studies were conducted on animals or in vitro. Evidence from the few epidemiological studies is inconsistent, showing both an inverse or linear association in men between the consumption of flavonoids found in fruits and PD risk. No studies on non-flavonoid polyphenols were found.

Two prospective cohort studies were examined. The first one was a 22-year cohort study by Gao et al. The authors investigated the relationship between the consumption of flavonoids and the risk of developing PD [116]. Men who were in the highest quintile for the consumption of flavonoids had a 40% lower risk of developing PD (95% CI: 0.43–0.83). Anthocyanins found in blueberries or strawberries achieved the best association with PD with a decreased risk of 33% in a pooled sample (*p*-value for trend <0.55) [116]. The second prospective cohort study consisted of 2388 men and 2136 women in Finland (Finnish Mobile Clinic Survey,41-year follow-up) and showed a positive association between berry and fruit consumption and increased risk of PD in men [72]. The intake of fruits and berries, between the highest and the lowest tertile, was associated with a RR of 3.67 (95% CI: 1.30–10.36; *p* = 0.60) for PD risk in men. The intake of fresh fruits was associated with a RR of 2.41 (95% CI: 1.01–5.77; *p* = 0.48). In contrast, among women, there were signs of an inverse association between the consumption of berries and the risk of PD (the highest vs. the lowest tertile, RR: 0.54; *p* = 0.02). These results did not support the hypothesis that the consumption of fruits containing flavonoids will play a protective role in the pathogenesis of PD in men. The consumption of vegetables was not associated with the risk of developing the disease in either men or women [72].

There was an agreement in results between different cohort studies and the meta-analyses confirming the beneficial neuro-protective effects of caffeine found in coffee and tea. The latter contains many important substances, other than caffeine, such as EGCG, lipids, amino acids, mineral substances, volatile compounds and other methylxanthines. These results were more confirmed by in vitro studies and experimental animal models where tea drinking was identified to reduce peroxidation and enhance antioxidative enzyme activity [106]. For other flavonoids such as anthocyanins, quercetin, and nobiletin, controversy in the results of the two epidemiological studies involving fruits and vegetables may be due to the presence of subclasses in flavonoids that exhibit differences in their chemical properties and their ability to cross the blood brain barrier [117]. Another limitation may be the presence of pesticides and herbicides that can reduce the neuroprotective effect of flavonoids [117] and the presence of confounding variables such as coffee, alcohol, and vitamin D status and the long follow-up time. A possible change in diet or a drop out of participants during the study period may have weakened the strength of the association [72].

### 3.4. Dietary Patterns

Investigating the associations between dietary patterns (DP) of a population and risk of PD is a more global approach than studying single food items or nutrients; food components work in synergy to affect different functions of the body.

The most studied DP, the Mediterranean pattern (Medi pattern), is often linked to beneficial health outcomes [118,119]. Specific individual dietary factors present in the Medi pattern are found to be protective against PD [120], and some compounds associated with higher PD risk are present in smaller amounts in the Medi pattern [9,117]. The Medi pattern, by modulating pathways that are related to the aging process in general, such as brain atrophy [121] and telomere length [122,123], as well as inflammation and oxidative stress [124,125], could explain the positive results related to PD risk.

Data from both cohorts, the NHS and the HPFS, showed that a healthy DP, the prudent dietary pattern (PDP) with greater intakes of plant food and fish and moderate intake of alcohol, significantly decreased PD risk by 22% (*p* = 0.04) when lowest quintile of adherence was compared to the highest quintile. The Western pattern tended to be positively associated with the risk of PD among never smokers but not among ever smokers, suggesting once again a potential protective effect of smoking [67]. Participants in the highest quintile of Alternate Healthy Eating Index (AHEI) and Alternate Mediterranean Diet Score (aMedi score) were 30% and 25% less likely to develop PD than those in the lowest quintile, respectively [126].

In a Finnish cohort study, when the DP of individuals was evaluated by a modified AHEI, the diet quality failed to predict PD [72]. This was also observed when food items were studied separately. Interestingly, the modified AHEI has shown to be associated with reduced incidence of cardiovascular disease and stroke in previous studies, so that, based on the authors comments, the lack of association might be due to modified dietary habits during the long follow-up period (41 years) [72].

The Hellenic Longitudinal Investigation of Aging and Diet (HELIAD) showed that each unit increase in Medi score was associated with a 2% decreased probability for prodromal PD (*p* < 0.001). Compared to participants in the lowest quartile of Mediterranean diet adherence, those in the highest quartile were associated with a 21% lower probability for prodromal PD [127,128].

A 4.5-year cohort study investigating the MIND (Mediterranean-Dash Diet Intervention for Neurodegenerative delay) diet, which is a combination of the Mediterranean diet and DASH (Dietary Approaches to Stop Hypertension) diet, showed, after adjustment for age, sex, smoking, total energy intake, BMI, and depressive symptoms, that higher MIND diet scores were associated with a decreased risk of parkinsonism (HR: 0.89; 95% CI: 0.83–0.96) and a slower rate of parkinsonism progression (*p* = 0.04). The Mediterranean diet was marginally associated with reduced disease progression (*p* = 0.06). The DASH diet, by contrast, was not associated with either outcome [129]. 

In line with these findings, a meta-analysis by Sofi et al. published in 2010 showed that a two-point increase in Mediterranean diet adherence determines a 13% reduction in the incidence of neurodegenerative disease including PD (RR: 0.87; 95% CI: 0.80–0.96) [130].

According to a report by Mc Carty et al. in 2001, prevalence rates of PD among Americans and Europeans tend to be relatively uniform; sub-Saharan black Africans, rural Chinese, and Japanese, whose diets are more vegan or vegetarian, have lower rates of PD [131]. The author suggested a protective effect of such pattern. Concerning vegan and vegetarian patterns, all reports so far were case-control studies, and no cohort studies were identified to investigate the impact of such DP on PD risk [132]. 

In summary, the prospective studies have overall been directed toward a beneficial effect of Medi pattern adherence. The method used to evaluate this pattern through different scores and the method used to diagnose PD could influence the conclusion of the studies. High intake of dietary fiber in the Medi pattern might affect constipation and therefore may mask early features of PD without necessarily interfering with PD pathogenic process per se [128].

As for vegetarian diet, more elaborate studies are needed to better elucidate the impact of this type of DP on PD risk.

### 3.5. Microbiota

The human gut hosts tens of trillions of microorganisms, including more than 1000 species of bacteria [133]. The gene repertoire of our gut bacteria contains 150 times more unique genes than the human genome [134].

In PD, gastrointestinal (GI) dysregulation is often observed several years before the disease is even detected. PD is associated with changes in gut microbiota and this dysbiosis may be the mechanism of neuroinflammation that leads to PD pathology [135]. Braak et al. hypothesized that the disease begins in the gut and spreads from gut to brain via the gut-brain axis [136]. The gut microbiome influences the enteric nervous system and, via the vagal nerve, the central nervous system [13].

The gut microbiota in PD patients is characterized by a decrease in taxonomic diversity, mainly beta diversity and fecal bacterial count [137]. In most studies, information related to alpha diversity was missed and if present, no significant difference was found between cases and controls [138] According to Petrov et al., PD patients are characterized by a significant difference in representation of 9 genera and 15 species of microorganisms [139]. In this review, a summary of microbiota changes which possibly predict the onset of PD will be discussed. This will include the five main phylae: *Firmicutes, Actinobacteria, Bacteroidetes, Verrucomicrobia,* and *Proteobacteria*, as well as their families, genus, and species. 

#### 3.5.1. Firmicutes

A review of different case-control studies revealed consistency in data at the phylum level. The Keshervazian et al. study was the only one to report a significant change of abundance in the *Firmicutes phylum* in PD patients compared to controls [140]. No studies reported any significant differences in the *Firmicutes* to *Bacteroidetes* ratio in PD patients [137].

Within the *Firmicutes phylum*, a decrease in the *Faecalibacterium* genus (*Clostridium leptum*), mainly species *Faecalibacterium prausnitzi,* in PD patients were found in several studies [140,141]. A decrease in the genera *Ruminococcus*, *Blautia, Dorea* [139], and *Coprococcus* (*Clostridium coccoides family*) were also reported [139,142] as well as reduced levels of *Lachnospiraceae* [140]. A reduced level of *Lachnospiraceae, Blautia*, and *Faecalalibacterium* are consistent with short chain fatty acid (SCFA) deficiency, mainly butyrate. These bacteria facilitate the degradation of cellulose and starch, which leads to the production of volatile short chain fatty acids, including acetate, propionate, and butyrate [143]. This could potentially explain the inflammation and microglial activation in the brain [89,144] and the gastrointestinal problems common in PD, such as leaky gut, constipation [145], and colonic inflammation [146].

The family of *Lactobacillaceae* was more abundant in PD patients compared to control patients [137]. This is in accordance with findings reported in five previous studies [139,140,147,148,149]. Receiver Operating Characteristic (ROC) analysis showed that *Lactobacillus* abundance could be considered a predictor for PD patients with an area under the curve (AUC) of 0.68 (95% CI: 0.58–0.79). The increase could be related to the frequent constipation of PD patients, since *Lactobacillus* is known to be increased in constipation-type irritable bowel syndrome (IBS) or decreased in diarrhea-type IBS [150]. In contrast, Unger et al. observed a reduction in this family [141] that is compatible with a reduced expression of occludin (a tight junction protein) and a morphologically altered intestinal epithelial barrier [151]. This difference in results between studies could be accounted for by differences in mean age, disease duration, as well as medication status. 

The family of *Enterococcoceae* (genus *Enterococcus*, order *Lactobacillales*) was more abundant in PD patients than in controls [142]. *Enterococcus* is well known to produce endotoxins and promote inflammation in human intestines [142]. *Enterococcus Faecalis (species)* produces extracellular superoxide and hydrogen peroxide that damage colonic epithelial cell DNA [152]. 

In the order of *Clostridiales*, the family of *Christensenellaceae* showed an increase in PD patients [139,147]. This family was found to be associated with an increase abundance of bacteria of the *Oscillospira* genus, found in the gut microbiota of PD patients [153]. It is also associated with a decrease in body weight and a higher risk for PD [154].

#### 3.5.2. Actinobacteria 

Within the *Actinobacteria* phyla, most of the studies revealed an increase in its abundance in PD patients compared to controls [142], associated with a significant increase in *Bifidobacteriaceae* family and *Bifidobacterium* genus (*p* < 0.01), [139,141]. Three studies reported an increase in the genus *Bifidobacterium* in PD patients [139,141,147]. This increase could be a mechanism of protection against the advance of the disease or deterioration of the general status [155].

#### 3.5.3. Bacteroidetes

Within the phylum of *Bacteroidetes*, the *Prevotellaceae* family was found to be reduced in PD patients [139], mainly in both genus *Prevotella copri* [139,156] and *Prevotella clara* [156]. In the study done by Hasegawa et al., this genus was the most reduced one and differed by 3.2-fold in PD patients compared to controls [149]. The *Prevotellaceae* family was also associated with the clinical score of PD severity [148]. A decrease in the *Prevotella* genus was correlated with the onset of the disease and used as a biomarker for the diagnosis of PD [148]. This genus is well-known to break down complex carbohydrates and to provide SCFAs as well as thiamine and folate as byproducts that promote a healthy intestinal environment. A decrease in *Prevotella* numbers is likely to result in reduced production of these important micronutrients, reduced production of essential vitamins, and impaired secretions of gut hormones [148]. However, in all studies, *Prevotella* genus abundance did not reach statistical difference between PD patients and controls. This may be due to the difference in sample size, inclusion criteria, and type of diets. 

#### 3.5.4. Verrucomicrobia

In this phylum, the *Akkermansia* genus increased, although not significantly, in PD patients [156]. *Akkermansia muciniphelia* is well known to improve the barrier function of the gut epithelium [156]. This genus uses mucus as an energy source, increasing the exposure of the gut epithelium to microbial antigens and hence producing an inflammatory effect [145]. Preliminary evidence also linked *Akkermansia* to multiple sclerosis [157]. It seems that a steady state *of Akkermansia* is of major importance for normal gut functioning [157]. In the study by Bedarf et al., the *Akkermansia* genus achieved a high prediction score for PD (AUC = 0.84, *p* < 0.0001) [156]. 

#### 3.5.5. Proteobacteria

The role of this phylum in PD patients is still unclear and needs further investigation. In the study by Unger et al., the bacterial family *Enterobacteriaceae* were more abundant in PD patients compared to controls (*p* < 0.01) [141]. However, this abundance did not differ between PD phenotypes (*p* = 0.506). In the study by Scheperjans et al., an increase in the abundance of *Enterobacteriaceae* family was reported in patients with postural instability and gait difficulty compared to tremor-dominant PD patients [148]. Forsyth et al. reported an increase in intestinal permeability in PD that correlated with staining for *Escherichia coli* and concluded the presence of a compromised intestinal barrier [145]. In the study of Li et al., the microbial composition at the genus level showed a significantly higher abundance of *Proteus*, *Escherichia-Shigella,* in PD patients compared to controls (*p* < 0.001) [142]. These genera are putative pathobionts that can produce endotoxins and promote inflammation in human intestines and provide an internal environment for PD development [152]. 

The only study to determine *Proteobacteria* of the genus *Ralstonia* was Keshavarzian et al., who conducted microbiota research on mucosal biopsies and fecal samples from 38 PD patients and 34 healthy controls [140]. *Proteobacteria* were significantly more abundant in the mucosa of PD patients compared to controls. This abundance could be contributing to neuroinflammation and immune activation in PD patients [140].

Investigating whether microbiota analysis could be used as a biomarker for PD declaration and pathogenesis is worthwhile. According to all reviewed studies, PD is associated with a change in gut microbiota, and this dysbiosis can lead to PD pathology. Discrepancy in studies results, according to phyla, families, and species, was present, although there was a general agreement on the absence of differences in the *Firmicutes/Bacteroidetes* ratio. Among the nine reviewed case-control studies, the study by Keshavarzian et al. was the only one to find a decrease in the abundance of *Firmicutes* at the phylum level in patients with PD [140]. This could be related to differences in research design and methods used between studies. At a higher taxonomic level, four studies showed a decrease in *Prevotellaceae* family, mainly the genus *Prevotella copri* and *P. clara* [139,141,148,156] in PD patients as well as a reduced level in several families, species and genus from the *Firmicutes* phylum, that are the biomarkers of gut diversity and symbiosis, such *as Lachnospiraceae, Ruminococcus*, and *Faecalibacterium prausnitzi,.* These microbiotas are well known to enhance the fermentation of cellulose, pectin, and starch as well as producing volatile SCFAs. For other *Firmicutes, Proteobacteria*, and *Verrucomicrobia*, there is an agreement between at least three study results according to the increase in the abundance in several families, genus, and species pertaining to these phyla such as *Lactobacillaceae (Firmicutes)* [135,139,147,149], *Christensenellaceae (Firmicutes)* [139,147], *E.coli (Proteobacteria)* [140,141,142,148], and *Akkermansia (Verrucomicrobia)* [156]. They are known to be associated with PD clinical parameters such as constipation, diarrhea, and inflammation [145,150]. They are also associated with a high prediction score for PD [139]. The increase of *Lactobacillaceae* could be linked to the presence of constipation induced by medication [150] as well as a decrease in ghrelin secretion in PD patients [141]. Ghrelin has been identified as a gut hormone that contributes to the maintenance and protection of normal nigrostriatal dopamine function. The increase of *Bifidobacterium* (genus) in PD patients [139,141,158] was also correlated to a protective action against PD symptoms progression [155].

However, the beneficial or harmful effects of these genera and species on PD onset and whether these species could be used as biomarkers for PD diagnosis are difficult to confirm. These species of different genus and families of different microbiota phylae include a high diversity of strains and subgenus differences. In addition, cross sectional case-control study is not sufficiently powerful to deduce a causal relationship between change in gut microbiota and PD onset. Recommendations to enhance and elucidate the temporal and causal relationships between gut microbiota and PD would be through cohort prospective studies, using a broader analysis of microbiome composition, with the same technique such as 16SrRNA sequencing and the same methods and materials (sample size, duration or declaration of PD, dietary pattern, and/or medication status). 

## 4. Conclusions

Based on the current review there is still conflicting evidence regarding the relationship between dietary factors and risk of PD. Some evidence was found for a potential neuroprotective effect of UA, PUFA, particularly N-3 PUFA and aLNA, caffeine, tea and beer consumption as well as Medi pattern. Non-heme iron, dairy products and SAFA, on the other hand, showed to be related to an increase risk of PD. Another important finding was the gender dependent association with PD, found in several studies. Dietary factors were more often associated with PD risk in men than women. This observation might be explained by underlying hormonal and genetic factors and/or the lower prevalence of PD in females; however, additional investigation is needed. 

Methodological differences exist among studies such as sample size, type of population (NHS and HPFS only included health professionals), study duration, and type/number of adjusted variables. Adjustment for smoking would be important because nicotine stimulates dopaminergic transmission in the brain and has been related to reduced PD incidence [32]. Not all studies have adjusted for that factor. Additional bias is related to dietary assessment, recall bias, and intra- and inter-investigator bias. 

Another limitation is the single nutrient approach. In many studies, nutrients were studied alone without investigating their biological interactions and their bioavailability in the gut. It will be a very difficult task to determine how nutrients interact with each other. Studying each nutrient alone and associating it with PD risk will not produce an evidence-based association. 

Investigating dietary patterns and their association with PD is an interesting approach; healthy dietary patterns contain a variety of nutrients acting in a synergistic way. The best example was the memory aging project (MAP) while studying the Mediterranean diet and its impact on PD pathogenesis [129].

An important topic when considering the influence of food or food components is the presence of contaminants, such as pesticides that may attenuate any possible association between food components and PD. This was tagged while investigating a possible relationship between dairy products and flavonoids and PD risk respectively [119]. Furthermore, effect of antioxidant vitamins, such as vitamin C and beta-carotene from food, may be confounded by presence of pesticides in fruits and vegetables masking the potential benefit of antioxidants. Hence, the presence of pesticides or other contaminants could increase the risk for PD or hide a protective effect of specific food constituents [117].

Moreover, genetic variations could interact with several investigated factors and modulate the association with PD risk. For example, level of homocysteine which is a potential neurotoxic compound is influenced by the polymorphism of the MTHFR gene, which may modulate the effect of folic acid on homocysteine concentration [159]. Future studies that consider genetic and environmental factors and the risk of PD will generate more consistent findings related to environmental factors and PD.

Studying microbiota and its impact on PD pathogenesis is a recent and promising topic. All studies were recent, dating from 2014 and onwards. Only case-control studies were found and reviewed. Several limitations existed due to the presence of different methodical approaches in analyzing microbiota such as real time quantitative PCR (qPCR), 16rRNA or 16ScDNA sequencing, or other metagenomic shotgun sequencing. Studies also had different methods and duration.

Nevertheless, this extensive review went through several recent prospective studies and meta-analysis that highlighted the impact of several environmental factors, including nutrients and types of diets and their association with PD development. Further, well-designed prospective studies are recommended taking into consideration the main covariates. It is also worthy to develop studies that could explain further the observed gender differences and the underlying mechanisms.

## Supplementary Materials

The following are available online at https://www.mdpi.com/2072-6643/11/8/1896/s1, Table S1: Dietary fat intake and PD risk, Table S2: Vitamins intake and PD risk, Table S3: Minerals intake and PD risk, Table S4: Uric Acid level and PD risk, Table S5: Dairy product intake and PD risk, Table S6: Consumption of alcoholic beverages and PD risk, Table S7: Coffee, tea and other polyphenols containing food items and PD risk, Table S8: Dietary patterns and PD risk, Table S9: Microbiota and PD prevalence.

## References

[1] Van Den Eeden S.K., Tanner C.M., Bernstein A.L., Fross R.D., Leimpeter A., Bloch D.A., Nelson L.M. (2003). Incidence of Parkinson’s disease: Variation by age, gender, and race/ethnicity. Am. J. Epidemiol..

[2] De Lau L.M.L., Koudstaal P.J., Witteman J.C.M., Hofman A., Breteler M.M.B. (2006). Dietary folate, vitamin B12, and vitamin B6 and the risk of Parkinson disease. Neurology.

[3] Kalia L.V., Lang A.E. (2015). Parkinson’s disease. Lancet.

[4] Roy Sarkar S., Banerjee S. (2019). Gut microbiota in neurodegenerative disorders. J. Neuroimmunol..

[5] Doria M., Maugest L., Moreau T., Lizard G., Vejux A. (2016). Contribution of cholesterol and oxysterols to the pathophysiology of Parkinson’s disease. Free Radic. Biol. Med..

[6] Liu J.-P., Tang Y., Zhou S., Toh B.H., McLean C., Li H. (2010). Cholesterol involvement in the pathogenesis of neurodegenerative diseases. Mol. Cell. Neurosci..

[7] Arokiasamy P., Uttamacharya U., Jain K., Biritwum R.B., Yawson A.E., Wu F., Guo Y., Maximova T., Espinoza B.M., Rodríguez A.S. (2015). The impact of multimorbidity on adult physical and mental health in low- and middle-income countries: What does the study on global ageing and adult health (SAGE) reveal?. BMC Med..

[8] Bousquet M., Calon F., Cicchetti F. (2011). Impact of omega-3 fatty acids in Parkinson’s disease. Ageing Res. Rev..

[9] Erro R., Brigo F., Tamburin S., Zamboni M., Antonini A., Tinazzi M. (2018). Nutritional habits, risk, and progression of Parkinson disease. J. Neurol..

[10] Fan C., Zirpoli H., Qi K. (2013). n-3 fatty acids modulate adipose tissue inflammation and oxidative stress. Curr. Opin. Clin. Nutr. Metab. Care.

[11] Kamel F., Goldman S.M., Umbach D.M., Chen H., Richardson G., Barber M.R., Meng C., Marras C., Korell M., Kasten M. (2014). Dietary fat intake, pesticide use, and Parkinson’s disease. Parkinsonism Relat. Disord..

[12] Chen H., Zhang S.M., Hernán M.A., Willett W.C., Ascherio A. (2003). Dietary intakes of fat and risk of Parkinson’s disease. Am. J. Epidemiol..

[13] De Lau L.M.L., Bornebroek M., Witteman J.C.M., Hofman A., Koudstaal P.J., Breteler M.M.B. (2005). Dietary fatty acids and the risk of Parkinson disease: The Rotterdam study. Neurology.

[14] Kyrozis A., Ghika A., Stathopoulos P., Vassilopoulos D., Trichopoulos D., Trichopoulou A. (2013). Dietary and lifestyle variables in relation to incidence of Parkinson’s disease in Greece. Eur. J. Epidemiol..

[15] Dong J., Beard J.D., Umbach D.M., Park Y., Huang X., Blair A., Kamel F., Chen H. (2014). Dietary fat intake and risk for Parkinson’s disease. Mov. Disord..

[16] Abbott R.D., Ross G.W., White L.R., Sanderson W.T., Burchfiel C.M., Kashon M., Sharp D.S., Masaki K.H., Curb J.D., Petrovitch H. (2003). Environmental, life-style, and physical precursors of clinical Parkinson’s disease: Recent findings from the Honolulu-Asia aging study. J. Neurol..

[17] Tan L.C., Methawasin K., Tan E.-K., Tan J.H., Au W.-L., Yuan J.-M., Koh W.-P. (2016). Dietary cholesterol, fats and risk of Parkinson’s disease in the Singapore Chinese health study. J. Neurol. Neurosurg. Psychiatr..

[18] Wang A., Lin Y., Wu Y., Zhang D. (2015). Macronutrients intake and risk of Parkinson’s disease: A meta-analysis. Geriatr. Gerontol. Int..

[19] Zhang Y., Chen J., Qiu J., Li Y., Wang J., Jiao J. (2015). Intakes of fish and polyunsaturated fatty acids and mild-to-severe cognitive impairment risks: A dose-response meta-analysis of 21 cohort studies 1–3. Am. J. Clin. Nutr..

[20] Marion-Letellier R., Savoye G., Ghosh S. (2015). Polyunsaturated fatty acids and inflammation. IUBMB Life.

[21] Zhang S.M.M., Hernán M.A., Chen H., Spiegelman D., Willett W.C.C., Ascherio A., Spiegelman D., Hernan M.A., Chen H., Willett W.C.C. (2002). Intakes of vitamins E and C, carotenoids, vitamin supplements, and PD risk. Neurology.

[22] Yang F., Wolk A., Håkansson N., Pedersen N.L., Wirdefeldt K. (2017). Dietary antioxidants and risk of Parkinson’s disease in two population-based cohorts. Mov. Disord..

[23] Selhub J., Troen A., Rosenberg I.H. (2010). B vitamins and the aging brain. Nutr. Rev..

[24] Bonetti F., Brombo G., Zuliani G. (2016). The relationship between hyperhomocysteinemia and neurodegeneration. Neurodegener. Dis. Manag..

[25] Yasui K., Kowa H., Nakaso K., Takeshima T., Nakashima K. (2000). Plasma homocysteine and MTHFR C677T genotype in levodopa-treated patients with PD. Neurology.

[26] Chen H., Zhang S.M., Schwarzschild M.A., Hernán M.A., Logroscino G., Willett W.C., Ascherio A. (2004). Folate intake and risk of Parkinson’s disease. Am. J. Epidemiol..

[27] Lee E.S.Y., Chen H., Soliman K.F.A., Charlton C.G. (2005). Effects of homocysteine on the dopaminergic system and behavior in rodents. Neurotoxicology.

[28] De Lau L.M.L., Koudstaal P.J., Van Meurs J.B.J., Uitterlinden A.G., Hofman A., Breteler M.M.B. (2005). Methylenetetrahydrofolate reductase C677T genotype and PD. Ann. Neurol..

[29] Dalto D.B., Matte J.J. (2017). Pyridoxine (Vitamin B6) and the glutathione peroxidase system; a link between one-carbon metabolism and antioxidation. Nutrients.

[30] Segura-Aguilar J. (2015). A new mechanism for protection of dopaminergic neurons mediated by astrocytes. Neural Regen. Res..

[31] Parra M., Stahl S., Hellmann H. (2018). Vitamin B6 and its role in cell metabolism and physiology. Cells.

[32] Ma C., Liu Y., Neumann S., Gao X. (2017). Nicotine from cigarette smoking and diet and Parkinson disease: A review. Transl. Neurodegener..

[33] Dias V., Junn E., Mouradian M.M. (2013). The role of oxidative stress in Parkinson’s disease. J. Parkinsons Dis..

[34] Zhao X., Zhang M., Li C., Jiang X., Su Y., Zhang Y. (2019). Benefits of vitamins in the treatment of Parkinson’s disease. Oxid. Med. Cell. Longev..

[35] Borrelli E., Chambon P. (1999). Control of transcription and neurological diseases. Mol. Psychiatr..

[36] Paganini-Hill A. (2001). Risk factors for Parkinson’s disease: The leisure world cohort study. Neuroepidemiology.

[37] Hughes K.C., Schwarzschild M.A., Gao X., Weisskopf M.G., Ascherio A. (2016). Intake of antioxidant vitamins and risk of Parkinson’s disease. Mov. Disord..

[38] Etminan M., Gill S.S., Samii A. (2005). Intake of vitamin E, vitamin C, and carotenoids and the risk of Parkinson’s disease: A meta-analysis. Lancet Neurol..

[39] Takeda A., Nyssen O.P., Syed A., Jansen E., Bueno-De-Mesquita B., Gallo V. (2014). Vitamin A and carotenoids and the risk of Parkinson’s disease: A systematic review and meta-analysis. Neuroepidemiology.

[40] Clapham D.E. (2007). Calcium signaling. Cell.

[41] Surmeier D.J., Schumacker P.T., Guzman J.D., Ilijic E., Yang B., Zampese E. (2017). Calcium and Parkinson’s disease. Biochem. Biophys. Res. Commun..

[42] Chen H., Zhang S.M., Hernán M.A., Willett W.C., Ascherio A. (2002). Diet and Parkinson’s disease: A potential role of dairy products in men. Ann. Neurol..

[43] Park M., Ross G.W., Petrovitch H., White L.R., Masaki K.H., Nelson J.S., Tanner C.M., Curb J.D., Blanchette P.L., Abbott R.D. (2005). Consumption of milk and calcium in midlife and the future risk of Parkinson disease. Neurology.

[44] Ward R.J., Zucca F.A., Duyn J.H., Crichton R.R., Zecca L. (2014). The role of iron in brain ageing and neurodegenerative disorders. Lancet Neurol..

[45] Dexter D.T., Wells F.R., Lees A.J., Agid F., Agid Y., Jenner P., Marsden C.D. (1989). Increased nigral iron content and alterations in other metal ions occurring in brain in Parkinson’s disease. J. Neurochem..

[46] Gorell J.M., Johnson C.C., Rybicki B.A., Peterson E.L., Kortsha G.X., Brown G.G., Richardson R.J. (1997). Occupational exposures to metals as risk factors for Parkinson’s disease. Neurology.

[47] Logroscino G., Gao X., Chen H., Wing A., Ascherio A. (2008). Dietary iron intake and risk of Parkinson’s disease. Am. J. Epidemiol..

[48] Barnham K.J., Bush A.I. (2008). Metals in Alzheimer’s and Parkinson’s diseases. Curr. Opin. Chem. Biol..

[49] Cicero C.E., Mostile G., Vasta R., Rapisarda V., Signorelli S.S., Ferrante M., Zappia M., Nicoletti A. (2017). Metals and neurodegenerative diseases. A systematic review. Environ. Res..

[50] Cheng P., Yu J., Huang W., Bai S., Zhu X., Qi Z., Shao W., Xie P. (2015). Dietary intake of iron, zinc, copper, and risk of Parkinson’s disease: A meta-analysis. Neurol. Sci..

[51] Davies K.J.A., Sevanian A., Muakkassah-Kelly S.F., Hochstein P. (1986). Uric acid-iron ion complexes. A new aspect of the antioxidant functions of uric acid. Biochem. J..

[52] Crotty G.F., Ascherio A., Schwarzschild M.A. (2017). Targeting urate to reduce oxidative stress in Parkinson disease. Exp. Neurol..

[53] Vitart V., Rudan I., Hayward C., Gray N.K., Floyd J., Palmer C.N.A., Knott S.A., Kolcic I., Polasek O., Graessler J. (2008). SLC2A9 is a newly identified urate transporter influencing serum urate concentration, urate excretion and gout. Nat. Genet..

[54] Church W.H., Ward V.L. (1994). Uric acid is reduced in the substantia nigra in Parkinson’s disease: Effect on dopamine oxidation. Brain Res. Bull..

[55] Huang T.T., Hao D.L., Wu B.N., Mao L.L., Zhang J. (2017). Uric acid demonstrates neuroprotective effect on Parkinson’s disease mice through Nrf2-ARE signaling pathway. Biochem. Biophys. Res. Commun..

[56] De Lau L.M.L., Koudstaal P.J., Hofman A., Breteler M.M.B. (2005). Serum uric acid levels and the risk of Parkinson disease. Ann. Neurol..

[57] Chen H., Mosley T.H., Alonso A., Huang X. (2009). Plasma urate and Parkinson’s disease in the atherosclerosis risk in communities (ARIC) study. Am. J. Epidemiol..

[58] Weisskopf M.G., O’Reilly E., Chen H., Schwarzschild M.A., Ascherio A. (2007). Plasma urate and risk of Parkinson’s disease. Am. J. Epidemiol..

[59] Gao X., Chen H., Choi H.K., Curhan G., Schwarzschild M.A., Ascherio A. (2008). Diet, urate, and Parkinson’s disease risk in men. Am. J. Epidemiol..

[60] Shen L., Ji H.-F. (2013). Low uric acid levels in patients with Parkinson’s disease: Evidence from meta-analysis. BMJ Open.

[61] Wen M., Zhou B., Chen Y.H., Ma Z.L., Gou Y., Zhang C.L., Yu W., Jiao L. (2017). Serum uric acid levels in patients with Parkinson’s disease: A meta-analysis. PLoS ONE.

[62] Alonso A., Rodríguez L.A.G., Logroscino G., Hernán M.A. (2007). Gout and risk of Parkinson disease: A prospective study. Neurology.

[63] O’Reilly E.J., Gao X., Weisskopf M.G., Chen H., Schwarzschild M.A., Spiegelman D., Ascherio A., Schwarzschild M.A., Gao X., Weisskopf M.G. (2010). Plasma urate and Parkinson’s disease in women. Am. J. Epidemiol..

[64] Jain S., Ton T.G.G., Boudreau R.M.M., Yang M., Thacker E.L.L., Studenski S., Longstreth W.T., Strotmeyer E.S.S., Newman A.B.B., Yang M. (2011). The risk of Parkinson disease associated with urate in a community-based cohort of older adults. Neuroepidemiology.

[65] Gao X., O’Reilly É.J., Schwarzschild M.A., Ascherio A. (2016). Prospective study of plasma urate and risk of Parkinson disease in men and women. Neurology.

[66] Ascherio A., LeWitt P., Xu K., Eberly S., Watts A., Matson W., Marras C., Kieburtz K., Rudolph A., Bogdanov M. (2009). Urate predicts rate of clinical decline in Parkinsons disease. Arch. Neurol..

[67] Abbott R.D., Ross G.W., Petrovitch H., Masaki K.H., Launer L.J., Nelson J.S., White L.R., Tanner C.M. (2016). Midlife milk consumption and substantia nigra neuron density at death. Neurology.

[68] Tanner C.M., Kame F., Ross G.W., Hoppin J.A., Goldman S.M., Korell M., Marras C., Bhudhikanok G.S., Kasten M., Chade A.R. (2011). Rotenone, paraquat, and Parkinson’s disease. Environ. Health Perspect..

[69] Wirdefeldt K., Adami H.-O.O., Cole P., Trichopoulos D., Mandel J. (2011). Epidemiology and etiology of Parkinson’s disease: A review of the evidence. Eur. J. Epidemiol..

[70] Chen H., O’Reilly E., McCullough M.L., Rodriguez C., Schwarzschild M.A., Calle E.E., Thun M.J., Ascherio A. (2007). Consumption of dairy products and risk of Parkinson’s disease. Am. J. Epidemiol..

[71] Hughes K.C., Gao X., Kim I.Y., Wang M., Weisskopf M.G., Schwarzschild M.A., Ascherio A., Hughes K.C., Schwarzschild M.A., Ascherio A. (2017). Intake of dairy foods and risk of Parkinson disease. Neurology.

[72] Sääksjärvi K., Knekt P., Lundqvist A., Männistö S., Heliövaara M., Rissanen H., Järvinen R. (2013). A cohort study on diet and the risk of Parkinson’s disease: The role of food groups and diet quality. Br. J. Nutr..

[73] Jiang W., Ju C., Jiang H., Zhang D. (2014). Dairy foods intake and risk of Parkinson’s disease: A dose-response meta-analysis of prospective cohort studies. Eur. J. Epidemiol..

[74] Bellou V., Belbasis L., Tzoulaki I., Evangelou E., Ioannidis J.P.A. (2016). Environmental risk factors and Parkinson’s disease: An umbrella review of meta-analyses. Parkinsonism Relat. Disord..

[75] Ascherio A., Schwarzschild M.A. (2016). The epidemiology of Parkinson’s disease: Risk factors and prevention. Lancet Neurol..

[76] Kistner A., Krack P. (2014). Parkinson’s disease: No milk today?. Front. Neurol..

[77] Kamel F., Tanner C., Umbach D., Hoppin J., Alavanja M., Blair A., Comyns K., Goldman S., Korell M., Langston J. (2007). Pesticide exposure and self-reported Parkinson’s disease in the agricultural health study. Am. J. Epidemiol..

[78] Hernán M.A., Logroscino G., Rodríguez L.A.G. (2004). A prospective study of alcoholism and the risk of Parkinson’s disease. J. Neurol..

[79] Palacios N., Gao X., O’Reilly E., Schwarzschild M., McCullough M.L., Mayo T., Gapstur S.M., Ascherio A.A. (2012). Alcohol and risk of Parkinson’s disease in a large, prospective cohort of men and women. Mov. Disord..

[80] Tan L.C., Koh W.-P., Yuan J.-M., Wang R., Au W.-L., Tan J.H., Tan E.-K., Yu M.C. (2008). Differential effects of black versus green tea on risk of Parkinson’s disease in the Singapore Chinese health study. Am. J. Epidemiol..

[81] Hernán M.A., Chen H., Schwarzschild M.A., Ascherio A. (2003). Alcohol consumption and the incidence of Parkinson’s disease. Ann. Neurol..

[82] Liu R., Guo X., Park Y., Wang J., Huang X., Hollenbeck A., Blair A., Chen H. (2013). Alcohol consumption, types of alcohol, and Parkinson’s disease. PLoS ONE.

[83] Eriksson A.-K., Löfving S., Callaghan R.C., Allebeck P. (2013). Alcohol use disorders and risk of Parkinson’s disease: Findings from a Swedish national cohort study 1972–2008. BMC Neurol..

[84] Sääksjärvi K., Knekt P., Männistö S., Lyytinen J., Jääskeläinen T., Kanerva N., Heliövaara M. (2014). Reduced risk of Parkinson’s disease associated with lower body mass index and heavy leisure-time physical activity. Eur. J. Epidemiol..

[85] Noyce A.J., Bestwick J.P., Silveira-Moriyama L., Hawkes C.H., Giovannoni G., Lees A.J., Schrag A. (2012). Meta-analysis of early nonmotor features and risk factors for Parkinson disease. Ann. Neurol..

[86] Zhang D., Jiang H., Xie J. (2014). Alcohol intake and risk of Parkinson’s disease: A meta-analysis of observational studies. Mov. Disord..

[87] Collins M.A., Neafsey E.J., Mukamal K.J., Gray M.O., Parks D.A., Das D.K., Korthuis R.J. (2009). Alcohol in moderation, cardioprotection, and neuroprotection: Epidemiological considerations and mechanistic studies. Alcohol. Clin. Exp. Res..

[88] Choi H.K., Curhan G. (2004). Beer, liquor, and wine consumption and serum uric acid level: The third national health and nutrition examination survey. Arthritis Care Res..

[89] Gaffo A.L., Roseman J.M., Jacobs D.R., Lewis C.E., Shikany J.M., Mikuls T.R., Jolly P.E., Saag K.G. (2010). Serum urate and its relationship with alcoholic beverage intake in men and women: Findings from the Coronary Artery Risk Development in Young Adults (CARDIA) cohort. Ann. Rheum. Dis..

[90] Ghiselli A., Natella F., Guidi A., Montanari L., Fantozzi P., Scaccini C. (2000). Beer increases plasma antioxidant capacity in humans. J. Nutr. Biochem..

[91] Nishioka K., Sumida T., Iwatani M., Kusumoto A., Ishikura Y., Hatanaka H., Yomo H., Kohda H., Ashikari T., Shibano Y. (2002). Influence of moderate drinking on purine and carbohydrate metabolism. Alcohol. Clin. Exp. Res..

[92] Yamamoto T., Moriwaki Y., Takahashi S., Tsutsumi Z., Ka T., Fukuchi M., Hada T. (2002). Effect of beer on the plasma concentrations of uridine and purine bases. Metab. Clin. Exp..

[93] Ames B.N., Cathcart R., Schwiers E., Hochstein P. (1981). Uric acid provides an antioxidant defense in humans against oxidant- and radical-caused aging and cancer: A hypothesis. Proc. Natl. Acad. Sci. USA.

[94] Jones D.C., Gunasekar P.G., Borowitz J.L., Isom G.E. (2000). Dopamine-induced apoptosis is mediated by oxidative stress and Is enhanced by cyanide in differentiated PC12 cells. J. Neurochem..

[95] Duan W., Ladenheim B., Cutler R.G., Kruman I.I., Cadet J.L., Mattson M.P. (2002). Dietary folate deficiency and elevated homocysteine levels endanger dopaminergic neurons in models of Parkinson’s disease. J. Neurochem..

[96] Jin F., Wu Q., Lu Y.-F., Gong Q.-H., Shi J.-S. (2008). Neuroprotective effect of resveratrol on 6-OHDA-induced Parkinson’s disease in rats. Eur. J. Pharmacol..

[97] Khan M.M., Ahmad A., Ishrat T., Khan M.B., Hoda M.N., Khuwaja G., Raza S.S., Khan A., Javed H., Vaibhav K. (2010). Resveratrol attenuates 6-hydroxydopamine-induced oxidative damage and dopamine depletion in rat model of Parkinson’s disease. Brain Res..

[98] Jardim F.R., de Rossi F.T., Nascimento M.X., da Silva Barros R.G., Borges P.A., Prescilio I.C., de Oliveira M.R. (2018). Resveratrol and brain mitochondria: A review. Mol. Neurobiol..

[99] Kolahdouzan M., Hamadeh M.J. (2017). The neuroprotective effects of caffeine in neurodegenerative diseases. CNS Neurosci. Ther..

[100] Nakaso K., Ito S., Nakashima K. (2008). Caffeine activates the PI3K/Akt pathway and prevents apoptotic cell death in a Parkinson’s disease model of SH-SY5Y cells. Neurosci. Lett..

[101] Ross G.W., Abbott R.D., Petrovitch H., Morens D.M., Grandinetti A., Tung K.H., Tanner C.M., Masaki K.H., Blanchette P.L., Curb J.D. (2000). Association of coffee and caffeine intake with the risk of Parkinson disease. JAMA.

[102] Hu G., Bidel S., Jousilahti P., Antikainen R., Tuomilehto J. (2007). Coffee and tea consumption and the risk of Parkinson’s disease. Mov. Disord..

[103] Ascherio A., Weisskopf M.G., O’Reilly E.J., McCullough M.L., Calle E.E., Rodriguez C., Thun M.J. (2004). Coffee consumption, gender, and Parkinson’s disease mortality in the cancer prevention study II cohort: The modifying effects of estrogen. Am. J. Epidemiol..

[104] Ascherio A., Zhang S.M., Hernán M.A., Kawachi I., Colditz G.A., Speizer F.E., Willett W.C. (2001). Prospective study of caffeine consumption and risk of Parkinson’s disease in men and women. Ann. Neurol..

[105] Costa J., Lunet N., Santos C., Santos J., Vaz-Carneiro A. (2010). Caffeine exposure and the risk of Parkinson’s disease: A systematic review and meta-analysis of observational studies. J. Alzheimers Dis..

[106] Kujawska M., Jodynis-Liebert J. (2018). Polyphenols in Parkinson’s disease: A systematic review of in vivo studies. Nutrients.

[107] Caruana M., Vassallo N. (2015). Tea polyphenols in Parkinson’s disease. Adv. Exp. Med. Biol..

[108] Pan T., Jankovic J., Le W. (2003). Potential therapeutic properties of green tea polyphenols in Parkinson’s disease. Drugs Aging.

[109] Kyoung Chun O., Jin Chung S., Song W.O. (2007). The journal of nutrition nutrient requirements and optimal nutrition estimated dietary flavonoid intake and major food sources of U.S. adults. J. Nutr..

[110] Chow H.H.S., Cai Y., Hakim I.A., Crowell J.A., Shahi F., Brooks C.A., Dorr R.T., Hara Y., Alberts D.S. (2003). Pharmacokinetics and safety of green tea polyphenols after multiple-dose administration of epigallocatechin gallate and polyphenon E in healthy individuals. Clin. Cancer Res..

[111] Li F.J., Ji H.F., Shen L. (2012). A meta-analysis of tea drinking and risk of Parkinson’s disease. Sci. World J..

[112] Moosavi F., Hosseini R., Saso L., Firuzi O. (2015). Modulation of neurotrophic signaling pathways by polyphenols. Drug Des. Dev. Ther..

[113] Angeloni C., Malaguti M., Barbalace M.C., Hrelia S. (2017). Bioactivity of olive oil phenols in neuroprotection. Int. J. Mol. Sci..

[114] Bendini A., Cerretani L., Alegria C.-P., Ana Maria G.-C., Antonio S.-C., Alberto F.-G., Lercker G. (2007). Phenolic molecules in virgin olive oils: A survey of their sensory properties, health effects, antioxidant activity and analytical methods. An overview of the last decade. Molecules.

[115] Beauchamp G.K., Keast R.S., Morel D., Lin J., Pika J., Han Q., Lee C.H., Smith A.B., Breslin P.A. (2005). Phytochemistry: Ibuprofen-like activity in extra-virgin olive oil. Nature.

[116] Gao X., Cassidy A., Schwarzschild M.A., Rimm E.B., Ascherio S.A. (2012). Habitual intake of dietary flavonoids and risk of Parkinson disease. Neurology.

[117] Agim Z.S., Cannon J.R. (2015). Dietary factors in the etiology of Parkinson’s disease. Biomed. Res. Int..

[118] Bach-Faig A., Berry E.M., Lairon D., Reguant J., Trichopoulou A., Dernini S., Medina F.X., Battino M., Belahsen R., Miranda G. (2011). Mediterranean diet pyramid today. Science and cultural updates. Public Health Nutr..

[119] Whayne T.F. (2014). Ischemic heart disease and the Mediterranean diet. Curr. Cardiol. Rep..

[120] Mohammad-Beigi H., Aliakbari F., Sahin C., Lomax C., Tawfike A., Schafer N.P., Amiri-Nowdijeh A., Eskandari H., Møller I.M., Hosseini-Mazinani M. (2019). Oleuropein derivatives from olive fruit extracts reduce—Synuclein fibrillation and oligomer toxicity. J. Biol. Chem..

[121] Gu Y., Brickman A.M., Stern Y., Habeck C.G., Razlighi Q.R., Luchsinger J.A., Manly J.J., Schupf N., Mayeux R., Scarmeas N. (2015). Mediterranean diet and brain structure in a multiethnic elderly cohort. Neurology.

[122] Crous-Bou M., Fung T.T., Prescott J., Julin B., Du M., Sun Q., Rexrode K.M., Hu F.B., De Vivo I. (2014). Mediterranean diet and telomere length in nurses’ health study: Population based cohort study. BMJ.

[123] Wang H., Chen H., Gao X., McGrath M., Deer D., De Vivo I., Schwarzschild M.A., Ascherio A. (2008). Telomere length and risk of Parkinson’s disease: Telomere length and Parkinson’s disease. Mov. Disord..

[124] Barnham K.J., Masters C.L., Bush A.I. (2004). Neurodegenerative diseases and oxidative stress. Nat. Rev. Drug Discov..

[125] Chrysohoou C., Panagiotakos D.B., Pitsavos C., Das U.N., Stefanadis C. (2004). Adherence to the Mediterranean diet attenuates inflammation and coagulation process in healthy adults: The ATTICA study. J. Am. Coll. Cardiol..

[126] Gao X., Chen H., Fung T.T., Logroscino G., Schwarzschild M.A., Hu F.B., Ascherio A. (2007). Prospective study of dietary pattern and risk of Parkinson disease. Am. J. Clin. Nutr..

[127] Anastasiou C.A., Yannakoulia M., Kosmidis M.H., Dardiotis E., Hadjigeorgiou G.M., Sakka P., Arampatzi X., Bougea A., Labropoulos I., Scarmeas N. (2017). Mediterranean diet and cognitive health: Initial results from the Hellenic longitudinal investigation of ageing and diet. PLoS ONE.

[128] Maraki M.I., Yannakoulia M., Stamelou M., Stefanis L., Xiromerisiou G., Kosmidis M.H., Dardiotis E., Hadjigeorgiou G.M., Sakka P., Anastasiou C.A. (2018). Mediterranean diet adherence is related to reduced probability of prodromal Parkinson’s disease. Mov. Disord..

[129] Agarwal P., Wang Y., Buchman A.S., Holland T.M., Bennett D.A., Morris M.C. (2018). MIND diet associated with reduced incidence and delayed progression of Parkinsonism in old age. J. Nutr. Health Aging.

[130] Sofi F., Abbate R., Gensini G.F., Casini A. (2010). Accruing evidence on benefits of adherence to the Mediterranean diet on health: An updated systematic review and meta-analysis. Am. J. Clin. Nutr..

[131] McCarty M.F. (2001). Does a vegan diet reduce risk for Parkinson’s disease?. Med. Hypotheses.

[132] Okubo H., Miyake Y., Sasaki S., Murakami K., Tanaka K., Fukushima W., Kiyohara C., Tsuboi Y., Yamada T., Oeda T. (2012). Dietary patterns and risk of Parkinson’s disease: A case-control study in Japan. Eur. J. Neurol..

[133] Falony G., Joossens M., Vieira-Silva S., Wang J., Darzi Y., Faust K., Kurilshikov A., Bonder M.J., Valles-Colomer M., Vandeputte D. (2016). Population-level analysis of gut microbiome variation. Science.

[134] Qin J., Li R., Raes J., Arumugam M., Solvsten Burgdorf K., Manichanh C., Nielsen T., Pons N., Levenez F., Yamada T. (2010). A human gut microbial gene catalog established by metagenomic sequencing Europe PMC funders group. Nature.

[135] Hopfner F., Künstner A., Müller S.H., Künzel S., Zeuner K.E., Margraf N.G., Deuschl G., Baines J.F., Kuhlenbäumer G. (2017). Gut microbiota in Parkinson disease in a northern German cohort. Brain Res..

[136] Braak H., Del Tredici K. (2008). Nervous system pathology in sporadic Parkinson disease. Neurology.

[137] Gerhardt S., Mohajeri M.H. (2018). Changes of colonic bacterial composition in Parkinson’s disease and other neurodegenerative diseases. Nutrients.

[138] Cryan J.F., Dinan T.G. (2012). Mind-altering microorganisms: The impact of the gut microbiota on brain and behaviour. Nat. Rev. Neurosci..

[139] Petrov V.A., Saltykova I.V., Zhukova I.A., Alifirova V.M., Zhukova N.G., Dorofeeva Y.B., Tyakht A.V., Kovarsky B.A., Alekseev D.G., Kostryukova E.S. (2017). Analysis of gut microbiota in patients with parkinson’s disease. Bull. Exp. Biol. Med..

[140] Keshavarzian A., Green S.J., Engen P.A., Voigt R.M., Naqib A., Forsyth C.B., Mutlu E., Shannon K.M. (2015). Colonic bacterial composition in Parkinson’s disease. Mov. Disord..

[141] Unger M.M., Spiegel J., Dillmann K.U., Grundmann D., Philippeit H., Bürmann J., Faßbender K., Schwiertz A., Schäfer K.H. (2016). Short chain fatty acids and gut microbiota differ between patients with Parkinson’s disease and age-matched controls. Parkinsonism Relat. Disord..

[142] Li W., Wu X., Hu X., Wang T., Liang S., Duan Y., Jin F., Qin B. (2017). Structural changes of gut microbiota in Parkinson’s disease and its correlation with clinical features. Sci. Chin. Life Sci..

[143] Topping D.L., Clifton P.M. (2001). Short-chain fatty acids and human colonic function: Roles of resistant starch and nonstarch polysaccharides. Physiol. Rev..

[144] Sampson T.R., Debelius J.W., Thron T., Janssen S., Shastri G.G., Ilhan Z.E., Challis C., Schretter C.E., Rocha S., Gradinaru V. (2016). Gut Microbiota regulate motor deficits and neuroinflammation in a model of Parkinson’s disease. Cell.

[145] Forsyth C.B., Shannon K.M., Kordower J.H., Voigt R.M., Shaikh M., Jaglin J.A., Estes J.D., Dodiya H.B., Keshavarzian A. (2011). Increased intestinal permeability correlates with sigmoid mucosa alpha-synuclein staining and endotoxin exposure markers in early Parkinson’s disease. PLoS ONE.

[146] Segain J.P., Raingeard de la Blétière D., Bourreille A., Leray V., Gervois N., Rosales C., Ferrier L., Bonnet C., Blottière H.M., Galmiche J.P. (2000). Butyrate inhibits inflammatory responses through NFkappaB inhibition: Implications for Crohn’s disease. Gut.

[147] Hill-Burns E.M., Debelius J.W., Morton J.T., Wissemann W.T., Lewis M.R., Wallen Z.D., Peddada S.D., Factor S.A., Molho E., Zabetian C.P. (2017). Parkinson’s disease and Parkinson’s disease medications have distinct signatures of the gut microbiome. Mov. Disord..

[148] Scheperjans F., Aho V., Pereira P.A.B., Koskinen K., Paulin L., Pekkonen E., Haapaniemi E., Kaakkola S., Eerola-Rautio J., Pohja M. (2015). Gut microbiota are related to Parkinson’s disease and clinical phenotype. Mov. Disord..

[149] Hasegawa S., Goto S., Tsuji H., Okuno T., Asahara T., Nomoto K., Shibata A., Fujisawa Y., Minato T., Okamoto A. (2015). Intestinal dysbiosis and lowered serum lipopolysaccharide-binding protein in Parkinson’s disease. PLoS ONE.

[150] Calkwood J., Vollmer T., Fox R.J., Zhang R., Novas M., Sheikh S.I., Viglietta V. (2016). Safety and tolerability of delayed-release dimethyl fumarate administered with interferon beta or glatiramer acetate in relapsing-remitting multiple sclerosis. Int. J. MS Care.

[151] Clairembault T., Leclair-Visonneau L., Coron E., Bourreille A., Le Dily S., Vavasseur F., Heymann M.F., Neunlist M., Derkinderen P. (2015). Structural alterations of the intestinal epithelial barrier in Parkinson’s disease. Acta Neuropathol. Commun..

[152] Huycke M.M., Abrams V., Moore D.R. (2002). Enterococcus faecalis produces extracellular superoxide and hydrogen peroxide that damages colonic epithelial cell DNA. Carcinogenesis.

[153] Goodrich J.K., Waters J.L., Poole A.C., Sutter J.L., Koren O., Blekhman R., Beaumont M., Van Treuren W., Knight R., Bell J.T. (2014). Human genetics shape the gut microbiome. Cell.

[154] Cattaneo A., Cattane N., Galluzzi S., Provasi S., Lopizzo N., Festari C., Ferrari C., Guerra U.P., Paghera B., Muscio C. (2017). Association of brain amyloidosis with pro-inflammatory gut bacterial taxa and peripheral inflammation markers in cognitively impaired elderly. Neurobiol. Aging.

[155] Amaral W.Z., Lubach G.R., Proctor A., Lyte M., Phillips G.J., Coe C.L. (2017). Social influences on prevotella and the gut microbiome of young monkeys. Psychosom. Med..

[156] Bedarf J.R., Hildebrand F., Coelho L.P., Sunagawa S., Bahram M., Goeser F., Bork P., Wüllner U. (2017). Functional implications of microbial and viral gut metagenome changes in early stage L-DOPA-naïve Parkinson’s disease patients. Genome Med..

[157] Cantarel B.L., Waubant E., Chehoud C., Kuczynski J., Desantis T.Z., Warrington J., Venkatesan A., Fraser C.M., Mowry E.M. (2015). Gut microbiota in MS: Possible influence of immunomodulators. J. Investig. Med..

[158] Andrews Z.B., Erion D., Beiler R., Liu Z.-W., Abizaid A., Zigman J., Elsworth J.D., Savitt J.M., DiMarchi R., Tschop M. (2009). Ghrelin promotes and protects nigrostriatal dopamine function via a UCP2-dependent mitochondrial mechanism. J. Neurosci..

[159] Liew S.C., Gupta E. (2015). Das Methylenetetrahydrofolate Reductase (MTHFR) C677T polymorphism: Epidemiology, metabolism and the associated diseases. Eur. J. Med. Genet..

