# Supplementary material for: Nutritional Risk Factors, Microbiota and Parkinson’s Disease: What Is the Current Evidence?
Source: Nutrients. 2019 Aug 14;11(8):1896. doi: 10.3390/nu11081896 (PMC6722832; doi:10.3390/nu11081896)
Supplement: Supplementary file 1 [file nutrients-11-01896-s001.pdf]

Table S1: Dietary fat intake and PD risk:

Author	Study design	Population	Sample size	Factors studied	RR/OR- 95%CI	PD risk
**Chen et al, 2003 [12]**	Prospective cohort study	Health Professionals Follow-up Study (HPFS) and Nurses' Health Study (NHS)	47,331 men and 88,563 women, 191 men PD cases and 186 women PD cases	Fat intake	SAFA (men) : 1.83 (1.10, 3.03)	+
**Abbott et al, 2003 [16]**	Prospective cohort study	Honolulu-Asia Aging Study	8,006 participants, 137 cases	Environmental, lifestyle, and physical attributes	PUFAs (men who never smoked a cigarette) p=0.042 No OR available	-
**De Lau et al, 2005 [13]**	Prospective cohort study	The Rotterdam Study	5,289 participants, 51 PD cases	High intake of unsaturated fatty acids	Total fat: 0.69 (0.52, 0.91)<br>MUFAs: 0.68 (0.50, 0.94)<br>PUFAs: 0.66 (0.46, 0.96)	-<br>-<br>-
**Kyrozis et al, 2013 [14]**	Prospective cohort study	EPIC–Greece cohort	26,173 participants, 88 PD cases	Dietary and lifestyle variables	N-6 PUFA: 0.69 (0.50, 0.98)<br>LA: 0.79 (0.60,1.04)	-<br>-
**Dong et al, 2014 [15]**	Prospective cohort study	NIH-AARP Diet and Health Study	299,617 controls and 1,087 PD cases	Dietary fat intakes	n-6 PUFA: 1.23 (1.02, 1.49)	**NSR**
**Kamel et al, 2014 [22]**	Case-control nested within a cohort study	Farming and Movement Evaluation (FAME) nested in Agricultural Health Study (AHS)	336 controls and 89 PD cases	Dietary fat intake, altered response to environmental neurotoxicants	PUFAs: 0.4 (0.2, 0.8)<br>aLNA: 0.4 (0.2, 0.8)	-<br>-
**Kamel et al, 2014 [22]**	Meta-analysis study	9 studies, 3 case-controls and 6 prospective study	Not available	All categories of fat	aLNA: 0.81 (0.68,0.96)	-
**Tan et al, 2016 [17]**	Prospective cohort study	Singapore Chinese Health Study	63,257 participants, 411 PD cases	Dietary fat intakes	Cholesterol: 0.53 (0.33, 0.84)	-

					MUFAs (women): 0.44 (0.22, 0.88)	**-**
**Wang et al, 2015 [18]**	Meta-analysis study	Pertinent studies were identified by a search of PubMed and Embase	232, 869 participants, 1,482 PD cases	Macronutrients intake	Cholesterol: 0.97 (0.75, 1.26)<br>Fat intake: 0.88 (0.74, 1.06)	**NSR**<br>**NSR**
**Zhang et al, 2015 [19]**	Meta-analysis study	21 cohort studies	181,580 participants, 4,438 cases (263 mild cognitive impairment, 320 cognitive decline, 1864 dementia, 1332 Alzheimer disease, and 659 PD)	Fish and PUFA intake	8-g/d of PUFA intake: 0.9 (0.80, 0.99)	**-**

-Decreased risk of PD
+ Increased risk of PD
NSR No Significant Relation

Table S2: Vitamins intake and PD risk:

Author	Study design	Population	Sample size	Factors studied	RR/OR-95%CI	PD risk
**Paganini-Hill et al, 2001 [36]**	Case-control nested within a cohort study	Southern California	13,979 participants, 395 PD cases 2,320 controls	Vitamin C, A intake	No available OR	**NSR**
**Zhang et al, 2002 [21]**	Prospective cohort study	NHS and HPFS cohort	76890 women (NHS), 47337 men	Vitamin C, E and carotenoids intake	Vitamin E: 0.68 (0.49, 0.93)	**Higher intake of vitamin E: -**

			(HPFS)			
**Chen et al, 2004 [26]**	Prospective cohort study	NHS and HPFS cohort	47,341 men and 88,716 women, 248 PD men cases and 167 women PD cases	Vitamin B6, B9, B12 intake	Vitamin B6 : 1.0 (0.7, 1.4)<br>Vitamin B9 : 1.1 (0.8, 1.5)<br>Vitamin B12 : 0.8 (0.6, 1.1)	**NSR**
**Etminan et al, 2005 [38]**	Meta-analysis study	6 case control studies, 1 cohort study, 1 cross-sectional study	Not available	Beta-carotene, vitamin C, vitamin E	Vitamine E:<br>Moderate intake: 0.81 (0.67, 0.98)<br>High intake: 0.78 (0.57,1.06)<br>Vitamine C/beta-carotene	**-**<br><br><br><br><br>**NSR**
**De Lau et al, 2006 [2]**	Prospective cohort study	The Rotterdam Study	5,289 participants, 72 PD cases	Vitamin B6, B9, B12 intake	Vitamin B6 : 0.46 (0.22, 0.96)<br>Vitamin B9 : 0.75 (0.37, 1.49)<br>Vitamin B12 : 1.11 (0.61, 2.01)	**-**<br>**NSR**<br>**NSR**
**Takeda et al, 2013 [39]**	Meta-analysis study	8 studies (7 case-control and 1 cohort study)	Not available	Dietary intakes of vitamin A and carotenoids	Vitamin A: 1.09 (0.84, 1.42)<br>α-Carotene: 0.84 (0.59, 1.18)<br>β-Carotene: 0.91 (0.70, 1.20)<br>β-Cryptoxanthin: 0.96 (0.66, 1.40)<br>Lycopene: 1.03 (0.64, 1.65)	**NSR**
**Hughes et al, 2016 [37]**	Prospective cohort study	NHS and HPFS cohort	80,750 female (NHS)<br>48,672 men (HPFS)<br>1036 PD cases	Intakes of antioxidant, vitamins E and C and carotenoids	Vitamin E: 0.93 (0.75, 1.14)<br>Carotenoids: 0.97 (0.69, 1.37)<br>Vitamin C: 0.81	**NSR**<br>**NSR**<br>**NSR**

			(554 in HPFS and 482 in NHS)		(0.65, 1.01)	
**Yang et al, 2017 [22]**	Prospective cohort study	Swedish Mammography Cohort (SMC) and the Cohort of Swedish Men (COSM)	84,774 participants, 1,329 PD cases	Vitamin C, E, beta carotene and total antioxydant capacity intake	Beta carotene:<br>Men: 0.84 (0.84, 0.99)<br>Women: 0.86 (0.78, 0.95)<br>Vitamin E:<br>Women: 0.87 (0.79, 0.96)<br>Vitamin C:<br>Woman: 0.91 (0.83, 1.00)<br>Dietary total antioxidant:<br>Men: 1 (0.93, 1.07)<br>Woman: 0.93 (0.84, 1.02)	**-**<br>**-**<br>**NSR**<br>**NSR**

-Decreased risk of PD
+ Increased risk of PD
NSR No Significant Relation

Table S3: Minerals intake and PD risk:

**Author**	**Study design**	**Population**	**Sample size**	**Factors studied**	**RR/OR-95%CI**	**PD risk**
**Chen et al, 2002 [42]**	Prospective cohort study	NHS and HPFS cohort	47.331 men (HPF), 88563 women (NHS), 210 men PD cases, 184-woman PD cases	Food intake	Dairy calcium intake for men: 1.3 (1.0, 1.6)	**NSR**
**Park et al, 2005 [43]**	Prospective cohort study	Honolulu Heart Program	7,504 men, 128 PD cases	Milk and calcium intake in midlife	Dairy calcium intake, p= 0.046<br>Non-dairy calcium	+

					intake, p= 0.704<br>No available odd ratio	-
**Logroscino et al, 2008 [47]**	Prospective cohort study	NHS and HPFS cohort	47.406 men (HPFS), 76.947 women (NHS), 422 PD cases	Iron intake	Iron: 1.10 (0.74, 1.65)<br>High non-heme iron and low vitamin C intakes: 1.92 (1.14, 3.32)	**NSR**<br><br>+
**Cheng et al, 2015 [50]**	Meta-analysis study	1 cohort stud y(US) and 4 case control studies (3 US, 1 japan)	126,507 participants	Iron intake	Moderate dietary iron intake: 1.08 (0.61, 1.93)<br>high dietary iron intake: 1.03 (0.83, 1.30)	**NSR**

-Decreased risk of PD
+ Increased risk of PD
NSR No Significant Relation

Table S4: Uric Acid level and PD risk:

Author	Study design	Population	Sample size	Factors studied	RR/OR-95%CI	PD risk
**De Lau et al, 2005 [56]**	Prospective cohort study	The Rotterdam Study	4,695 participants, 68 PD cases	Plasma urate level	0.42 (0.18, 0.96)	**Higher plasma urate: -**
**Weisskopf et al, 2007 [58]**	Prospective cohort study	HPFS cohort	18,000 men, 84 PD cases	Plasma urate level	0.17 (0.04, 0.69)	**Higher plasma urate: -**
**Alonso et al, 2007 [62]**	Case-control nested within a cohort study	General Practice Research Database	6,634 controls and 1,052 PD cases	History of Gout	0.69 (0.48, 0.99)	**Gout: -**
**Gao et al, 2008 [59]**	Prospective cohort study	HPFS cohort	47,406 men	Diet that increases plasma urate level	0.47 (0.30, 0.74)	**Higher plasma urate: -**
**Chen et al, 2009 [57]**	Prospective cohort study	Atherosclerosis Risk in Communities (ARIC)	95 PD cases	Plasma urate level	0.4 (0.2, 0.8)	**Higher plasma urate: -**

**O'Reilly et al, 2010 [63]**	Case-control nested within a cohort study	NHS cohort	Cohort study:<br>5888-woman, 214 PD cases<br>Case-control:<br>105 PD cases, 518 controls	Plasma urate level	1.33 (0.69, 2.57)	**NSR**
**Jain et al, 2011 [64]**	Prospective cohort study	Cardiovascular Health Study (CHS)	5,749 participants, 214 PD cases	Plasma urate level	Urate > 500 µmol/l<br>Men: 1.55 (0.72–3.32)<br>Woman: 0.75 (0.18, 3.16)	**NSR**
**Shen et al, 2013 [60]**	Meta-analysis study	6 selected studies	1276 controls, 1217 PD cases	Serum uric acid level	No available odd ratio	**Lower serum uric acid: +**
**Gao et al, 2016 [65]**	Nested case-control study	3 ongoing US cohorts	1,267 control, 388 PD cases	Plasma urate level	Men: 0.63 (0.42, 0.95)<br>Woman: 1.04 (0.61, 1.78)	**Men: higher plasma urate: -**<br>**Woman: NSR**
**Wen et al, 2017 [61]**	Meta-analysis study	13 selected studies	2267 controls, 2379 PD cases	Serum uric acid level	Serum uric acid levels in PD patients were lower compared to sex and age-matched healthy controls (p<0.001)<br>No available odd ratio	**Lower serum uric acid: +**

-Decreased risk of PD
+ Increased risk of PD
NSR No Significant Relation

Table S5: Dairy product intake and PD risk:

**Author**	**Study design**	**Population**	**Sample size**	**Factors studied**	**RR/OR-95%CI**	**PD risk**
**Chen et al, 2002**	Prospective cohort	NHS and	51,529 men and	Food intake	Dairy intake:	

**[42]**	study	HPFS cohort	121,700 women, 210 PD men cases and 184 PD women cases		Men: 1.8 (1.2, 2.8)<br>Women: 1.1 (0.7, 1.7)	+<br>**NSR**
**Park et al, 2005 [43]**	Prospective cohort study	Honolulu Heart Program	7,504 men, 128 PD cases	Milk and calcium intake in midlife	2.6 (1.1,6.4)	+
**Chen et al, 2007 [70]**	Prospective cohort study	American Cancer Society's Cancer Prevention Study II Nutrition Cohort.	57,689 men and 73,175 women, 250 men PD cases and 138 women PD cases	Intake of dairy products	1.6 (1.1, 2.2)	+
**Kyrozis et al, 2013 [14]**	Prospective cohort study	EPIC–Greece cohort	26,173 participants, 120 PD cases	Dietary and lifestyle variables	Dairy total: 1.02 (0.84 1.26)<br>Milk: 1.19 (1.02, 1.38)<br>Yogurt: 0.77 (0.58, 1.02)	+<br>+<br>**NSR**
**Sääksjärvi et al, 2013 [72]**	Prospective cohort study	Finnish Mobile Clinic Health Examination Survey (FMC)	4524 participants, 85 PD cases	Food group and diet quality	Consumption of milk (highest tertile)for woman: 3.31 (1.10, 9.93)	+
**Jiang et al, 2014 [73]**	Meta-analysis study	Seven results from prospective studies	304,193 participants, 1,083 PD cases	Dairy foods	1.40 (1.20, 1.63)	+
**Abbott et al, 2016 [67]**	Prospective cohort study	Honolulu-Asia Aging Study	449 men participants, postmortem examinations	Midlife milk intake	0.82 (No available CI)	**NSR**
**Hughes et al, 2017 [71]**	Prospective cohort study	NHS and HPFS cohort	NHS: 80,736 participants<br>HPFS: 48,610<br>1,036 PD cases	Intake of dairy products	Low fat dairy: 1.34 (1.01, 1.79)	+

+ Increased risk of PD

NSR No Significant Relation

Table S6: Consumption of alcoholic beverages and PD risk:

Author	Study design	Population	Sample size	Factors studied	RR/OR-95%CI	PD risk
**Paganini-Hill, 2001 [36]**	Nested case-control study	Leisure World Laguna Hills, southern California	13,979 participants, 395 PD cases and 2,320 control	Etiologic factors	2+ alcoholic drinks: 0.77 (0.58, 1.03)	**-**
**Hernán et al, 2003 [81]**	Prospective cohort study	NHS and HPFS cohort	173,229 participants, 415 PD cases	Alcoholic beverages and another potentially habituating behaviour	Beer: 0.70 (0.5, 0.9)	**-**
**Hernán et al, 2004 [78]**	Case-control nested within a cohort study	General Practice Research Database (GPRD)	10,123 controls, 1,019 PD cases	Alcoholism	1.09 (0.67, 1.78)	**NSR**
**Kamel et al, 2007 [77]**	Case-control nested within a cohort study	Agricultural Health Study	55,931 participants, 78 PD cases	Pesticide exposure	1.10 (0.6, 1.8)	**NSR**
**Tan et al, 2008 [80]**	Prospective cohort study	Singapore Chinese Health Study	63,257 participants, 157 PD cases	Coffee, black tea, and green tea consumption	Drinkers: 0.58 (0.30, 1.11)	**NSR**
**Palacios et al, 2012 [79]**	Prospective cohort study	Cancer Prevention Study II Nutrition Cohort	132,403 participants, 605 PD cases	Alcohol consumption	Men consuming ≥30 grams alcohol/day: 1.29 (0.90, 1.86) Woman consuming ≥15 grams alcohol/day: 0.77 (0.41, 1.45)	**NSR**
**Noyce et al, 2012 [85]**	Meta-analysis study	22 selected studies	Not available	Association between PD and different risk factors	Alcohol: 0.9 (0.84, 0.96)	_
**Eriksson et al, 2013 [83]**	Prospective cohort study	Swedish National Inpatient Register	602,930 participants, 1,741 PD cases	Association between diagnosed alcohol use	1.38 (1.25, 1.53)	+

				disorders		
**Liu et al, 2013 [82]**	Prospective cohort study	NIH-AARP Diet and Health Study	306,895 participants, 1,113 PD cases	Total alcohol consumption, specific types of alcoholic beverage	Beer: 0.79 (0.68, 0.92)<br>Liquor: 1.35 (1.02, 1.80)	-<br>+
**Sääksjärvi et al, 2014 [84]**	Prospective cohort study	Finnish Mobile Clinic Health Examination Survey	6,715 participants, 101 PD cases	The prediction of various lifestyle factors	<5 g/day of alcohol intake: 1.94 (1.09, 3.47)	+
**Zhang et al, 2014 [86]**	Meta-analysis study	32 studies: 8 prospective, 17 matched case-control and 7 unmatched case-control involving	677,550 participants, 9994 PD cases	Alcohol consumption	Alcohol: 0.78 (0.67, 0.92)<br>Beer: 0.59 (0.39, 0.90)	-<br>-

-Decreased risk of PD

+ Increased risk of PD

NSR No Significant Relation

<u>Table S7: Coffee, tea and other polyphenols containing food items and PD risk:</u>

Author	Study design	Population	Sample size	Factors studied	RR/OR-95%CI	PD risk
**Ross et al, 2000 [101]**	Prospective cohort study	Honolulu Heart Program	8,006 Japanese-American men participants, 102 PD cases	Coffee and dietary caffeine intake	Higher coffee and caffeine intake decrease PD risk ($p < 0.001$)<br>No available odd ratio	-
**Ascherio et al, 2001 [104]**	Prospective cohort study	NHS and HPFS cohort	135,916 participants, 288 PD cases	Consumption of coffee	Men: 0.42 (0.23, 0.78)	-
**Ascherio et al, 2004 [103]**	Prospective cohort study	Cancer Prevention study II	Over 1 million participants, 909 PD men cases and 340 PD women cases	Caffeine consumption and gender difference	≥4 cups of coffee/day for women using postmenopausal estrogen : 0.47	-

					(0.27, 0.80)	
**Hu et al, 2007 [102]**	Prospective cohort study	Finnish population	29,335 participants, 200 PD cases	Coffee and tea consumption	1-4 cups of coffee/day: 0.53 (0.31, 0.92)<br>≥ 5 cups of coffee/day : 0.40 (0.23, 0.71)<br>≥ 3 cups of tea/day: 0.41 (0.20, 0.83)	**-**
**Tan et al, 2008 [80]**	Prospective cohort study	Singapore Chinese Health Study	63,257 participants, 157 PD cases	Coffee, black tea, and green tea consumption	Caffeine: 0.55 (95%: 0.35, 0.88)<br>Black tea: 0.29 (95%: 0.13, 0.67)	**-**<br>**-**
**Costa et al, 2010 [105]**	Meta-analysis study	26 studies:<br>7 cohort, 2 nested case-control, 16 case-control, and 1 cross-sectional study.	Not available	Caffeine/coffee consumption	300 mg increase in caffeine intake:<br>0.76 (0.72, 0.80)	**-**
**Li et al, 2012 [111]**	Meta-analysis study	8 articles published up to 2010	4250 controls, 1418 PD cases	Tea drinking	0.85 (0.74, 0.98)	**-**
**Gao et al, 2012 [116]**	Prospective cohort study	NHS and<br>HPFS cohort	129,617 participants, 805 PD cases	Flavonoid intake	Anthocyanins: 0.76 (0.61, 0.96)<br>Flavonoids: 0.77 (0.46, 1.28)	**-**<br>**NSR**
**Liu et al, 2012 [82]**	Prospective cohort study	National Institutes of Health (NIH)-AARP Diet and Health Study	304,980 participants, 940 PD cases	Caffeine intake	Men: 0.75 (0.60, 0.94)<br>Woman: 0.60 (0.39, 0.91)	**-**<br>**-**
**Sääksjärvi et al, 2013 [72]**	Prospective cohort study	Finnish Mobile Clinic Health<br>Examination Survey (FMC)	4524 participants, 85 PD cases	Food group or diet	Diet quality index:<br>Men: 1.83 (0.65, 5.18)<br>Woman: 0.97 (0.38, 2.48)	**NSR**

-Decreased risk of PD
+ Increased risk of PD
NSR No Significant Relation

Table S8: Dietary patterns and PD risk:

Author	Study design	Population	Sample size	Factors studied	RR/OR-95%CI	PD risk
**Gao et al, 2007 [126]**	Prospective cohort study	Health Professional Follow-up Study	131,368 participants, 508 PD cases	Dietary patterns	Mediterranean diet pattern: 0.75 (0.57, 1.00)	**-**
**Sofi et al, 2010 [130]**	Meta-analysis study	7 prospective studies	136,235 participants and 1074 PD cases	Effects of adherence to the Mediterranean diet on health status	0.87 (0.81, 0.94)	**-**
**Sääksjärvi et al, 2013 [72]**	Prospective cohort study	Finnish Mobile Clinic Health Examination Survey (FMC)	4524 participants, 85 PD cases	Food group and diet quality	Diet quality index:<br>Men: 1.83 (0.65, 5.18)<br>Woman: 0.97 (0.38, 2.48)	**NSR**
**Agarwal et al, 2018 [129]**	Prospective cohort study	Rush Memory and Aging Project (MAP)	706 participants, 302 PD cases	MIND diet	0.89 (0.83, 0.96)	**-**
**Maraki et al, 2018 [128]**	Prospective cohort study	HELIAD (Greece)	1,765 participants, 600 PD cases	Probability of prodromal PD and possible association with Mediterranean diet adherence	Adherence to the Mediterranean diet, p<0.001<br>No available odd ratio	**-**

-Decreased risk of PD
+ Increased risk of PD
NSR No Significant Relation

Table S9: Microbiota and PD prevalence:

Author	Study design	Population	Sample size	Factors studied	Microbiota variation	PD risk
**Scheperjans et al, 2015 [148]**	Case-control study	Hospital District of Helsinki and Uusimaa	72 control, 72 PD cases	Gut microbiota	*Prevotellaceae* Decreased by 77.6% **Increased content** of Enterobacteriaceae	+
**Hasegawa et al, 2015 [149]**	Case-control study	Outpatient clinic of Nagoya University Hospital and the Aichi Chapter of the Japan Parkinson's Disease Association	36 control, 52 PD cases	Gut microbiota	**Decreased content** *Clostridium coccoides* group, *Bacteroides fragilis* and *Prevotella* **Increased content** of *Lactobacillus*	+
**Keshavarzian et al, 2015 [140]**	Case-control study	Rush University Medical Center (RUMC; Chicago, IL)	34 control, 38 PD cases	Gut microbiota	**Decreased content** of *Faecalibacterium* **Increased content** *Proteobacteria*	+
**Unger et al, 2016 [141]**	Case-control study	German population	34 control, 34 PD cases	Gut microbiota	**Decreased content** of fecal SCFA concentrations *Bacteroidetes* and *Prevotellaceae* **Increased content** of *enterobacteriaceae, bifidobacterium genus*	+
**Hill-Burns et al, 2017 [147]**	Case-control study	NeuroGenetics Research Consortium in Seattle, Washington; Atlanta, Georgia; and	136 controls, 212 PD cases	Gut microbiota	**Increased content** of *Bifidobacteriaceae* and *Lactobacillaceae*	+

		Albany, New York				
**Hopfner et al, 2017 [135]**	Case-control study	German population	29 controls, 29 PD cases	Gut microbiota	**Increased content** of *Lactobacillaceae, Barnesiellaceae* and *Enterococcacea*	+
**Petrov et al, 2017 [139]**	Case-control study	Russian population	66 controls, 89 PD cases	Gut microbiota	**Decreased content** of *Bacteroides, Prevotella, Faecalibacterium* **Increased content** of *Lactobacillus, Bifidobacterium*	+
**Bedarf et al, 2017 [156]**	Case-control study	Department of Neurology at the University of Bonn	28 controls, 31 PD cases	Gut microbiota	**Decreased content** of *Prevotella copri* **Increased content** of *Akkermansia muciniphila* and unclassified *Firmicutes*	+
**Li et al, 2017 [142]**	Case-control study	Chinese population	14 controls, 24 PD cases		**Decreased content** of *Blautia, Faecalibacterium* and *Ruminococcus* **Increased content** *Escherichia-Shigella Streptococcus, Proteus* and *Enterococcus*	+

-Decreased risk of PD

+ Increased risk of PD

NSR No Significant Relation
